# PRMT6 acts as a pro-angiogenic factor in colorectal cancer

**DOI:** 10.1038/s41420-026-03256-y

**Published:** 2026-07-11

**Authors:** Haifeng Han, Xin Zhang, Mingxin Jin, Yali Chu, Fengjun Liu, Hui Qu, Cheng Chen

**Affiliations:** Department of General Surgery, Qilu Hospital of Shandong University, JiNan, China

**Keywords:** Tumour angiogenesis, Gastrointestinal cancer

## Abstract

The progression of colorectal cancer (CRC) is highly dependent on tumor angiogenesis, a process primarily regulated by hypoxia-inducible factor HIF-1α. This study focuses on the mechanistic role of protein arginine methyltransferase 6 (PRMT6) in CRC angiogenesis and reveals that PRMT6 is significantly overexpressed in CRC tissues, stabilizing HIF-1α via the autophagy-lysosome pathway. Specifically, PRMT6 catalyzes the asymmetric dimethylation of HIF-1α at arginine 463, which disrupts its interaction with the autophagy-related protein TAX1BP1, thereby preventing its degradation. In vivo experiments demonstrate that PRMT6 silencing reduces HIF-1α stability, decreases vascular endothelial growth factor A (VEGFA) expression, and markedly suppresses tumor angiogenesis and growth. This study identifies the PRMT6-HIF-1α axis as a novel therapeutic target for CRC and suggests that targeting this pathway may facilitate the development of precision anti-angiogenic therapies.

## Introduction

Tumor angiogenesis is pivotal in tumor progression, providing the essential oxygen and nutrients needed for tumor cell growth, invasion, and metastasis [1]. Under normal conditions, angiogenesis is tightly regulated during embryonic development, the menstrual cycle, and tissue repair. However, in pathological conditions such as cancer, this process becomes dysregulated, resulting in the excessive growth of abnormal blood vessels [2]. Given its critical role in tumor growth, anti-angiogenic therapy has emerged as a strategy in cancer treatment, with the goal of blocking new blood vessel formation, the tumor is deprived of essential nutrients and oxygen. While anti-angiogenic therapies have proven effective in certain tumor types, the complex regulatory network and the potential for increased drug resistance present significant challenges [3–6]. This highlights the need for a deeper understanding of the mechanisms underlying tumor angiogenesis.

Hypoxia-inducible factor 1-alpha (HIF-1α) plays a crucial role for the process of angiogenesis in tumors, particularly under the hypoxic conditions common in the tumor microenvironment. The regulatory mechanisms of HIF-1α involve oxygen-dependent degradation, post-translational modifications, nuclear translocation, and miRNA regulation, among others [7]. Under normal oxygen levels, HIF-1α undergoes ubiquitination and degradation mediated by the proteasomal protein VHL. However, in hypoxic conditions, it escapes degradation and translocates to the nucleus, where it forms an active complex with the HIF-1β subunit, regulating modulating the expression of downstream genes, including VEGFA, ANGPT2, and EphA1 [8–10]. Additionally, the autophagy-proteasome pathway is another key mechanism for HIF-1α degradation. Research has shown that lysosomal HSC70 and LAMP2A can interact with HIF-1α, leading to its lysosomal degradation [11, 12]. Additionally, Myo1b facilitates tumor advancement and angiogenesis in colorectal cancer by inhibiting the autophagic breakdown of HIF-1α [13]. Given that tumor tissues often exist in a hypoxic state, which hampers VHL-mediated ubiquitin-proteasome degradation of HIF-1α, enhancing the autophagy-lysosomal pathway for HIF-1α degradation could be an effective strategy to inhibit tumor angiogenesis.

The protein arginine methyltransferase 6 (PRMT6) is a protein arginine methyltransferase and a member of the PRMT family. It primarily catalyzes the mono-methylation and asymmetric di-methylation of arginine residues, playing a role in regulating various cellular processes such as transcription, translation, and signal transduction. PRMT6 is crucial in cancer, influencing various aspects of cellular behavior, including growth, migration, invasion, apoptosis, and drug resistance [14]. This makes it a potential target for anti-tumor therapies across multiple cancer types. The function of PRMT6 varies among different cancers. It can sometimes promote tumor progression and at other times inhibit tumor growth. For example, in prostate cancer, overexpression of PRMT6 can inhibit cell migration and invasion by upregulating TSP-1 and downregulating MMPs. In ovarian cancer, the expression level of PRMT6 is correlated with drug metabolism-related proteins G6PD and GSTP1, potentially leading to drug resistance [15, 16]. In colorectal cancer, PRMT6 facilitates tumor progression by epigenetically suppressing the expression of CDKN2B and CCNG1 [17]. We also prove that PRMT6 can regulate MYC signaling pathway in colorectal cancer [18]. However, it is unclear that the role of PRMT6 in colorectal cancer angiogenesis.

In this study, we revealed that PRMT6 positively regulates colorectal tumor angiogenesis. Mechanistically, we showed that knocking down PRMT6 inhibits HIF-1α/VEGFA signaling by inducing HIF-1α degradation through the autophagy-lysosome pathway. PRMT6-mediated asymmetric di-methylation (ADMA) of HIF-1α at arginine 463 prevents HIF-1α from interacting with the autophagy-associated protein TAX1BP1. Furthermore, we found that PRMT6-mediated tumor angiogenesis relies on HIF-1α R463 asymmetric di-methylation. In terms of clinical application, our findings demonstrated that silencing PRMT6 markedly inhibits tumor angiogenesis in vivo, indicating that PRMT6 may serve as a promising therapeutic target for colorectal cancer anti-angiogenesis treatment.

## Results

### PRMT6 might function as a pro-angiogenic gene in colorectal cancer

**Fig. 1:**
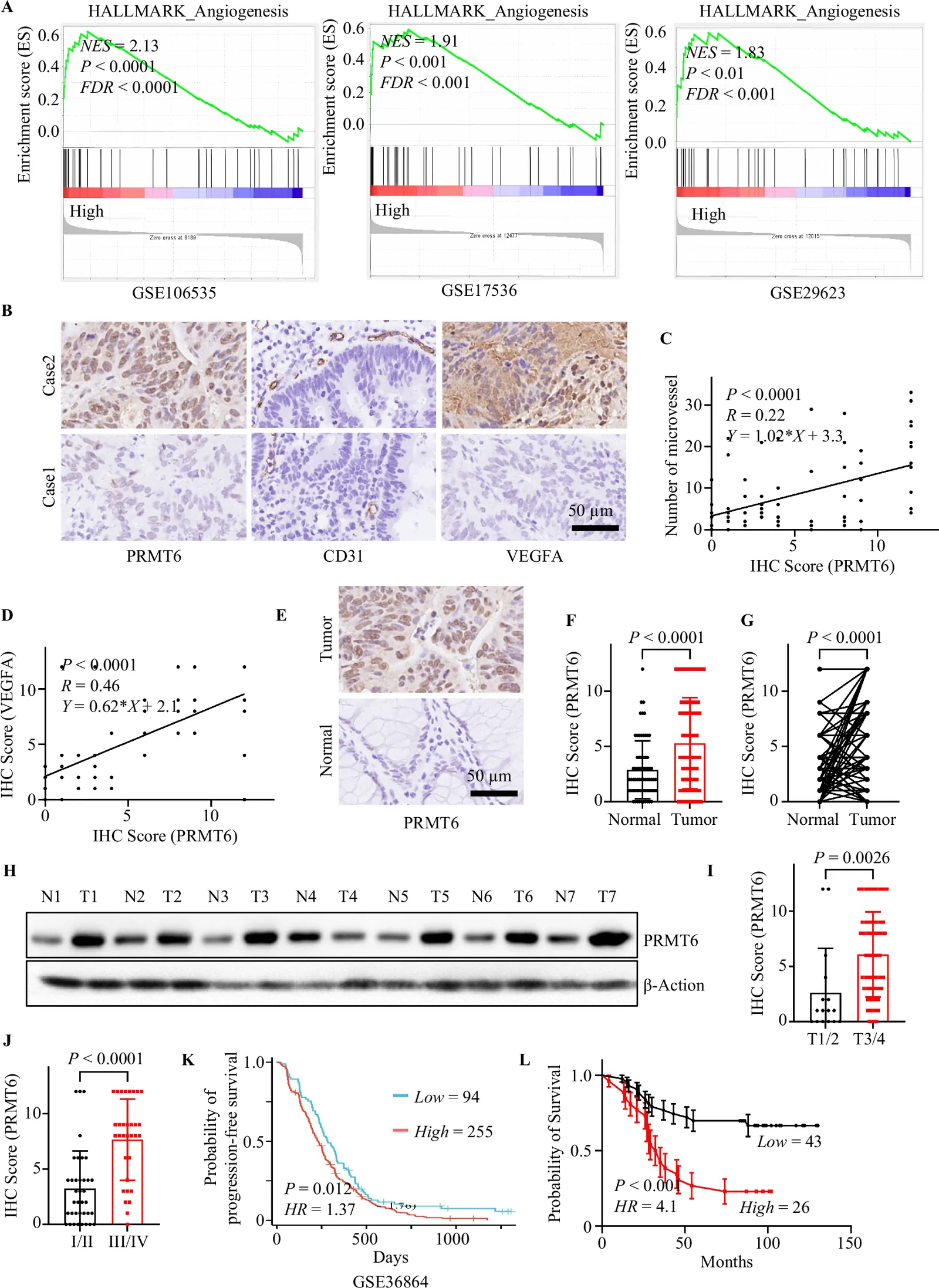
PRMT6 might act as a pro-angiogenesis factor in colorectal cancer. **A** GSEA analysis across multiple GEO gene sets. **B** Representative images of IHC staining. **C** Statistical analysis revealing the relationship between PRMT6 expression levels and the number of microvessels; *n* = 69; scale bar = 50 µm. **D** Statistical analysis showing the association between PRMT6 expression levels and VEGFA levels. E. Additional representative images of IHC staining; *n* = 69; scale bar = 50 µm. **F**, **G** Statistical analysis comparing PRMT6 expression in colorectal cancer tissues with adjacent normal colorectal tissues; *n* = 69. **H** Western blot analysis of PRMT6 expression in colorectal cancer and normal adjacent tissues; *n* = 7. **I**, **J** Statistical analysis of PRMT6 expression levels categorized by T stage and clinical stage; *n* = 69. **K** Kaplan-Meier survival analysis of GSE36864 patients based on PRMT6 expression levels; *n* = 349. **L** Kaplan-Meier survival analysis of colorectal cancer patients grouped according to PRMT6 expression levels; *n* = 69. Student’s t-test or the log-rank test was utilized for statistical analysis, and all western blots were performed in triplicate.

**Fig. 2:**
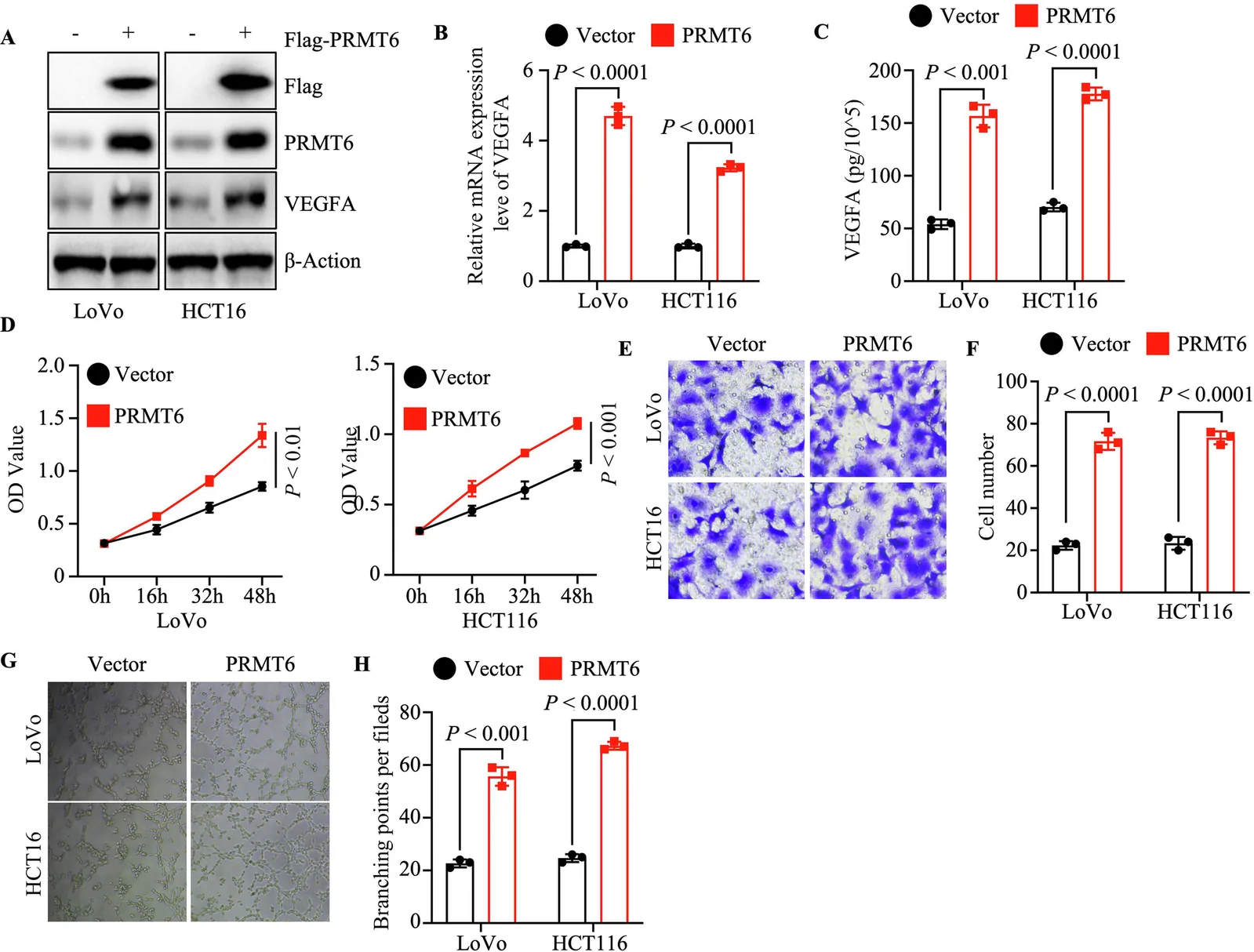
PRMT6 overexpression promoted tumor angiogenesis in vitro. **A** Western blot analyses to examine the expression of the specified protein. **B**, **C** RT-PCR and ELISA assays for detecting VEGFA levels in Vector and PRMT6 cancer cells; *n* = 3. **D** CCK8 assays to evaluate the proliferation potential of HUVECs treated with CM from Vector and PRMT6 cancer cells; *n* = 3. **E** Transwell assays to measure the migration capacity of HUVECs exposed to CM from Vector and PRMT6 cancer cells. **F** Statistical analysis for the results presented in (**E**); *n* = 3. **G** Tube formation assays to assess the ability of HUVECs to form tubes when cultured with CM from Vector and PRMT6 cancer cells. **H** Statistical analysis for the data shown in (**G**); *n* = 3. Student’s t-test was employed for the statistical evaluations, and all western blot experiments were performed in triplicate.

Given the crucial role of tumor angiogenesis and the unclear function of PRMT6 in colorectal tumor angiogenesis, we initially investigated whether PRMT6 regulates tumor angiogenesis through bioinformatics analysis. Notably, GSEA analysis revealed that the angiogenesis signaling pathway was more activated in tumors with high PRMT6 expression (Fig. 1A). We evaluated the expression levels of PRMT6 and VEGFA in 69 human colorectal tumors, using a CD31 antibody to identify intra-tumoral blood vessels. The results indicated a positive correlation between PRMT6 levels and both VEGFA expression and the number of intra-tumoral micro-vessels (Fig. 2B–D and Table 1). Additionally, analysis of the GEPIA database reinforced this positive association between PRMT6 and VEGFA across different cancer types (Fig. S1A).

**Table 1:** The clinicopathological characteristics.

Characteristics	Groups	Number (*n* = 69)	Percentage (%)
Age	< 60	39	56.5
	≥ 60	30	43.5
Gender	Male	37	53.6
	Female	32	46.4
T Stage	T1/2	16	23.2
	T3/4	53	76.8
N Stage	N0	38	55.1
	N1/2	31	44.9
M Stage	M0	59	85.5
	M1	10	14.5
Clinical Stage	I/II	36	52.2
	III/IV	33	47.8

To delve deeper into the role of PRMT6 in colorectal cancer, we measured its expression in 69 paired human colorectal tumors and adjacent normal tissues through immunohistochemistry (IHC). The analysis revealed that PRMT6 expression was significantly elevated in tumor tissues compared to their normal counterparts (Fig. 1E–G), which was further validated by Western blot assays (Fig. 1H). Furthermore, data from the TCGA-COAD/READ and GSE24550 datasets also showed heightened PRMT6 expression in tumor tissues compared to normal colorectal tissues (Fig. S1B–D). The GEPIA database analysis also demonstrated that PRMT6 was overexpressed in several cancer types (Fig. S1E). Receiver Operating Characteristic (ROC) analysis was performed to evaluate the diagnostic potential of PRMT6, where the Area Under the Curve (AUC) indicated the ability of the marker to distinguish CRC tissues from normal tissues. ROC analysis indicated that PRMT6 levels could serve as a useful diagnostic marker for colorectal cancer based on TCGA data (Fig. S1F). Moreover, increased PRMT6 expression was linked to advanced T stage (T3/T4) and clinical stage (III/IV) in tumors (Fig. 1I, J). Kaplan-Meier survival analysis revealed that colorectal cancer patients with higher PRMT6 expression had a significantly poorer overall survival (Fig. 1K, L), with similar trends noted in other cancer types (Fig. S2). Overall, these findings suggest that PRMT6 may act as an oncogene and promote angiogenesis in colorectal cancer.

### PRMT6 positively regulated colorectal tumor angiogenesis

To investigate the role of PRMT6 in tumor angiogenesis, we initially created stable LoVo and HCT116 cell lines expressing either a Vector or Flag-PRMT6 via lentiviral infection (Fig. 2A). Western blot analysis showed that overexpression of PRMT6 led to increased VEGFA levels (Fig. 2A), which was corroborated by RT-PCR and ELISA results (Fig. 2B, C). We then harvested CM from these cell lines to culture HUVECs. The data indicated that HUVECs treated with PRMT6 CM displayed enhanced proliferation compared to those treated with Vector CM (Fig. 2D). Additionally, HUVECs exposed to PRMT6 CM demonstrated greater migratory capacity than those cultured with Vector CM (Fig. 2E, F). Moreover, HUVECs in the presence of PRMT6 CM formed more complex tube structures than those treated with Vector CM (Fig. 2G, H).

**Fig. 3:**
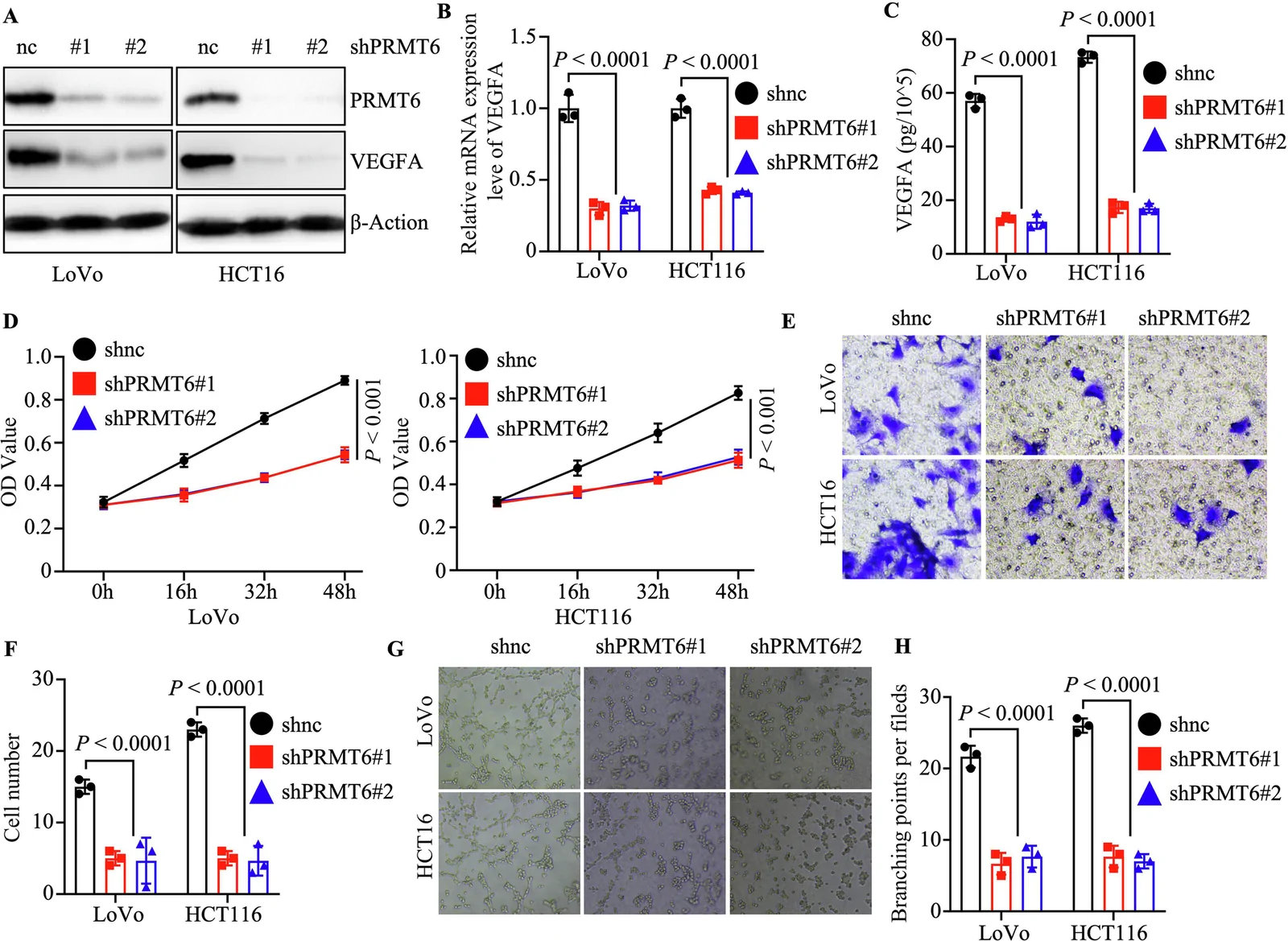
Silencing of PRMT6 inhibited tumor angiogenesis in vitro. **A** Western blot analyses to examine the expression levels of the specified protein. **B**, **C** RT-PCR and ELISA assays to quantify VEGFA expression in shNC and shPRMT6 cancer cells; *n* = 3. **D** CCK8 assays to evaluate the proliferation potential of HUVECs treated with CM from shNC and shPRMT6 cancer cells; *n* = 3. **E** Transwell assays to investigate the migration capability of HUVECs cultured with CM from shNC and shPRMT6 cancer cells. **F** Statistical analysis corresponding to the data illustrated in (**E**); *n* = 3. **G** Tube formation assays to assess the capacity of HUVECs to form tubes when exposed to CM from shNC and shPRMT6 cancer cells. **H** Statistical analysis for the findings shown in (**G**); *n* = 3. The Student’s t-test or one-way ANOVA analysis was applied for statistical assessments, and all western blot experiments were conducted in triplicate.

In contrast, we knocked down PRMT6 in LoVo and HCT116 cell lines using lentivirus. Western blot analysis confirmed that PRMT6 knockdown significantly reduced VEGFA expression (Fig. 3A–C). HUVECs cultured with shPRMT6 CM exhibited decreased proliferation, migration, and tube formation compared to those treated with shnc CM (Fig. 3D–H). Collectively, these results suggest that PRMT6 serves as a pro-angiogenic factor in colorectal cancer.

### PRMT6 positively regulated HIF1 signaling by blocking HIF1α autophagy-lysosome pathway degradation

**Fig. 4:**
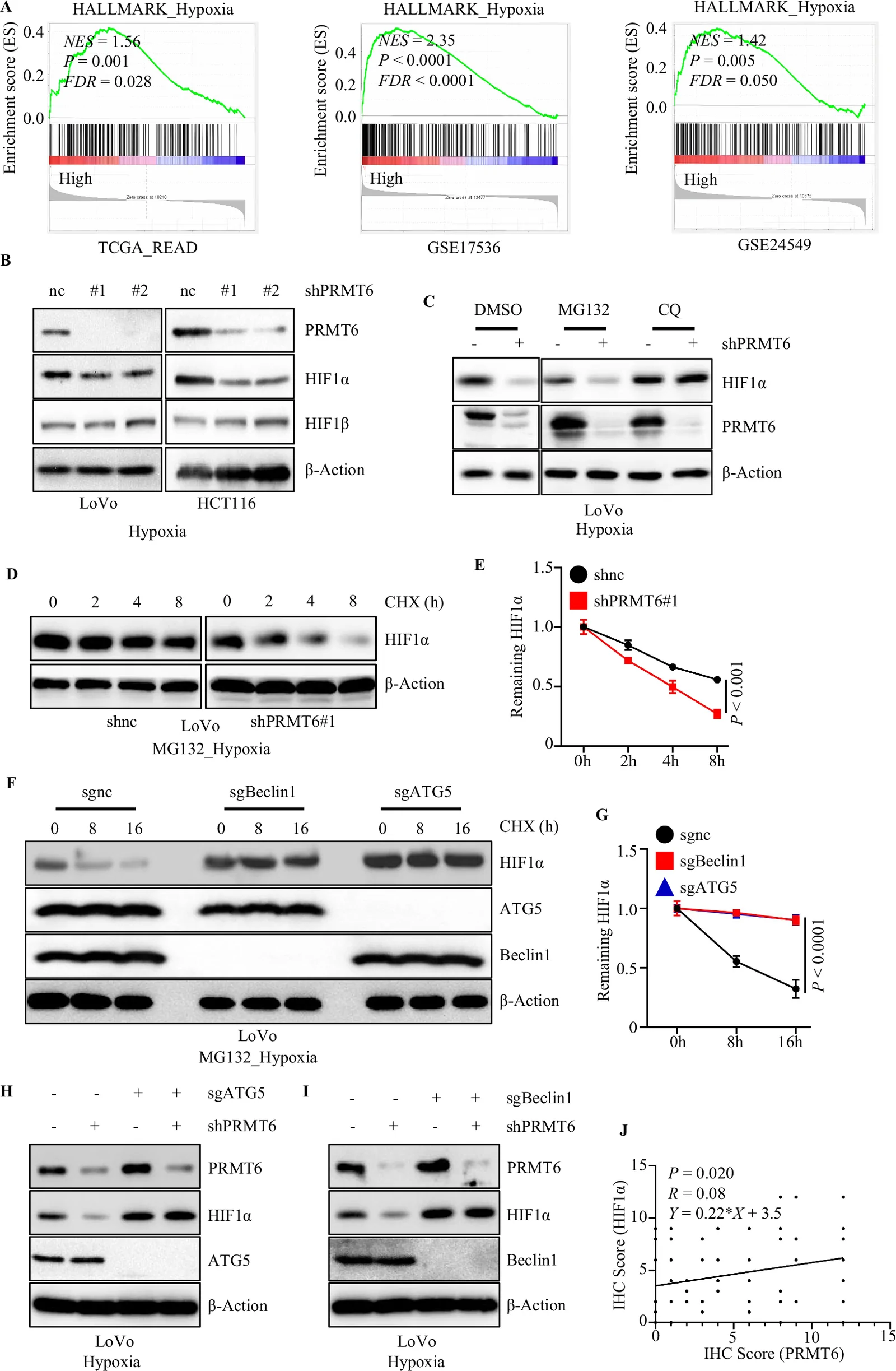
PRMT6 enhanced HIF1 signaling by preventing the degradation of HIF1α through the autophagy-lysosome pathway. **A** GSEA analysis performed on multiple GEO gene sets. **B** Western blot experiments to measure the levels of specific proteins in shNC and shPRMT6 cancer cells. **C** Following treatment of cancer cells with MG132 (10 μM, 8 h) or CQ (50 μM, 8 h), western blot analyses were conducted to evaluate the expression of targeted proteins in both shNC and shPRMT6 cancer cells. **D** Cells were treated with CHX (50 μg/mL) for the indicated time periods. To block proteasomal degradation, MG132 (10 μM) was added 1 h prior to CHX treatment and maintained throughout the experiment (total duration of 9 h). Representative western blot images of HIF-1α levels were shown. **E** Statistical analysis for Fig. 4D; *n* = 3. **F** Knocked out Beclin1 or ATG5 separately in LoVo cells, followed by western blots to assess the degradation rate of HIF1α in shNC and shPRMT6 cancer cells. **G** Statistical analysis for Fig. 4F; *n* = 3. **H**, **I** Individual knockout of Beclin1 or ATG5 was performed in LoVo cells, followed by western blotting to measure HIF1α expression levels in shNC and shPRMT6 cancer cells. **J** Statistical analysis was carried out to assess the relationship between PRMT6 and HIF1α expression levels; *n* = 69. Student’s t-test or one-way ANOVA analysis was utilized for statistical evaluation, and all western blot experiments were executed in triplicate.

To delve deeper into the mechanism by which PRMT6 influences angiogenesis in colorectal cancer, we performed Gene Set Enrichment Analysis (GSEA) using data from TCGA and several GEO datasets. The findings showed that tumors with elevated PRMT6 expression demonstrated enhanced activation of HIF1 signaling (Fig. 4A and Fig. S3A). Importantly, Western blot analysis indicated that knockdown of PRMT6 significantly lowered HIF1α expression, while HIF2α, HIF1β levels remained unchanged (Fig. 4B and Fig. S3B, C). We then ectopically expressed either wild-type PRMT6 (PRMT6 WT) or a PRMT6 enzyme activity-deficient mutant (PRMT6 KLA) in LoVo cancer cells and treated these cells with DMSO or the PRMT6 inhibitor PRMT6-IN-3. Western blot analysis showed that PRMT6 enzyme activity was crucial for PRMT6-mediated HIF1α expression (Fig. S3D, E). Interestingly, PRMT6 did not alter the mRNA levels of HIF1α (Figure S3F, G), indicating that PRMT6 modulates HIF1α expression at the post-translational level. Supporting this, treatment with chloroquine (CQ), an inhibitor of the autophagy-lysosome pathway, effectively prevented the decrease in HIF1α associated with shPRMT6. In contrast, MG132, which inhibits the ubiquitin-proteasome pathway, did not have the same effect (Fig. 4C). To further explore this, we used cycloheximide (CHX) to inhibit endogenous HIF1α synthesis and applied MG132 to block its degradation by the ubiquitin-proteasome pathway. Western blot analysis showed that PRMT6 knockdown accelerated HIF1α degradation (Fig. 4D, E). We also knocked out Beclin1 and ATG5, two proteins associated with autophagy, using the CRISPR-Cas9 system. Western blot analysis revealed that knockout of Beclin1 and ATG5 significantly inhibited the degradation of endogenous HIF1α (Fig. 4F, G) and counteracted the decrease in HIF1α caused by shPRMT6 (Fig. 4H, I). IHC assays conducted on 69 colorectal tumors confirmed a positive correlation between PRMT6 and HIF1α expression levels (Fig. 4J and Fig. S3H). Overall, these findings demonstrate that PRMT6 enhances HIF1 signaling by preventing the degradation of HIF1α through the autophagy-lysosome pathway.

### PRMT6 asymmetrically di-methylated of HIF1α at arginine 463

**Fig. 5:**
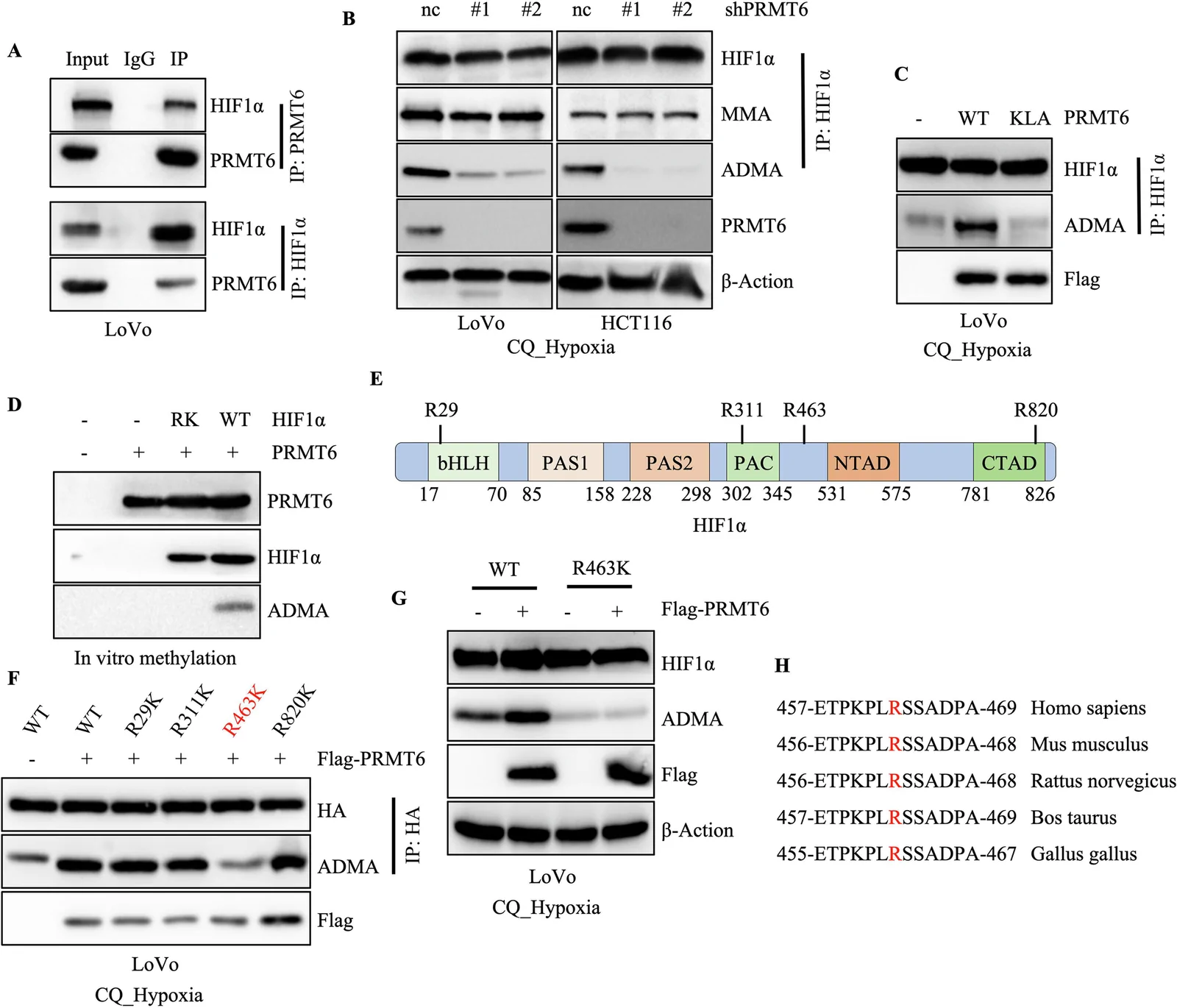
HIF1α arginine 463 was asymmetrically di-methylated by PRMT6. **A** Western blots and immunoblots were utilized to investigate the association between PRMT6 and HIF1α. **B** Western blots and immunoblots were executed to determine the MMA and ADMA levels of HIF1α in shNC and shPRMT6 cancer cells. **C** Western blots and immunoblots were conducted to analyze the ADMA levels of HIF1α in cancer cells that ectopically express either PRMT6 WT or the PRMT6 KLA mutant. **D** In vitro methylation assays to assess whether PRMT6 could directly asymmetrically dimethylate HIF1α. E. The potential arginine methylation sites of HIF1α shown. **F** LoVo cells were transfected with various HA-tagged HIF1α mutants and Flag-tagged PRMT6 plasmids. Western blots and immunoblots were performed to evaluate the ADMA levels of HIF1α. **G** Western blots and immunoblots were conducted to analyze the ADMA levels of HIF1α in various pre-treated cancer cells. **H** The amino acid sequence of HIF1α shown in various species. All Western blots were conducted in triplicate.

To further elucidate the mechanism behind PRMT6-mediated HIF1α expression, we first explored the GENEMANIA database, which suggested a potential interaction between PRMT6 and HIF1α (Fig. S4A). This interaction was confirmed by immunoprecipitation assays (Fig. 5A). Given PRMT6’s role as an arginine methyltransferase, we hypothesized that PRMT6 might regulate HIF1α through arginine methylation. Western blot and immunoprecipitation assays demonstrated that PRMT6 knockdown significantly reduced the asymmetrical di-methylation of HIF1α, but not its mono-methylation (Fig. 5B). Additionally, PRMT6 inhibitors also diminished HIF1α asymmetrical di-methylation (Fig. S4B). Consistent with this, overexpression of PRMT6 WT, but not the PRMT6 KLA mutant, increased the level of HIF1α asymmetrical di-methylation (Fig. 5C). Interestingly, hypoxia could increase the binding of PRMT6 and HIF1α, and the MMA level of HIF1α without affecting PRMT6 expression (Fig. S4C, D). In vitro methylation assays confirmed that PRMT6 could asymmetrically di-methylate HIF1α (Fig. 5D). Using GPS-MSP software, we identified four potential arginine methylation sites on HIF1α (R29, R311, R463, and R820) (Fig. 5E). We then constructed HIF1α mutants with these arginines replaced by lysines and transfected these mutants along with PRMT6 into LoVo cancer cells. Western blot and immunoprecipitation assays revealed that the R463K mutant abolished PRMT6-mediated HIF1α asymmetrical di-methylation (Fig. 5F). We also generated LoVo and HCT116 cancer cell lines stably expressing either HIF1α WT or HIF1α R463K using CRISPR-Cas9 and lentivirus (Fig. S4E, F). Western blot analysis further confirmed that the R463K mutant prevented PRMT6-mediated asymmetrical di-methylation of HIF1α (Fig. 5G). Furthermore, sequence analysis of HIF1α across multiple species showed that arginine 463 is conserved (Fig. 5H). Overall, our data indicate that PRMT6 asymmetrically di-methylates HIF1α at arginine 463.

### HIF1α R463 asymmetrical di-methylation inhibited its autophagy degradation by blocking the binding of HIF1α and TAX1BP1

**Fig. 6:**
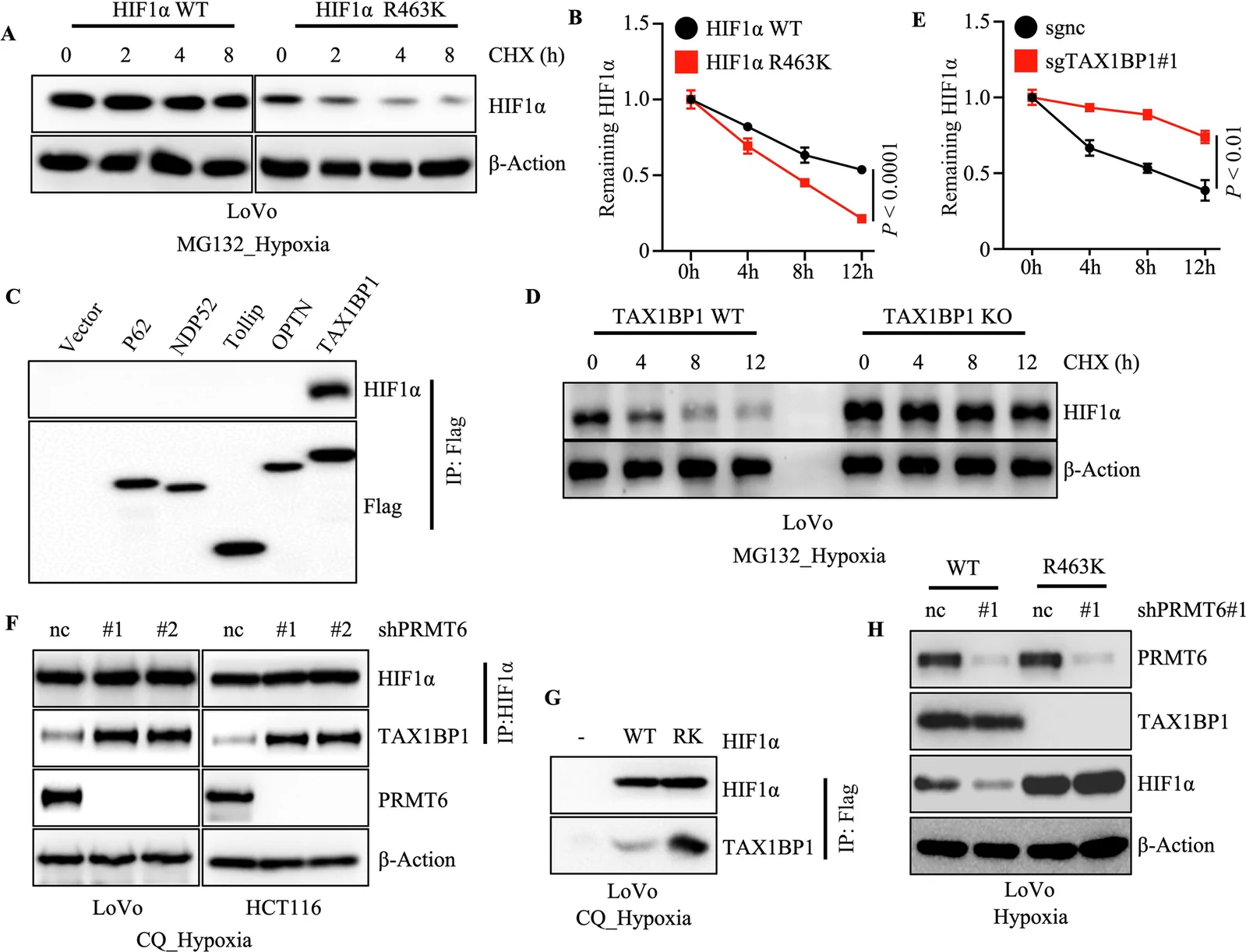
HIF1α R463 ADMA blocked its interaction with the autophagy-related protein TAX1BP1. **A** Cycloheximide (CHX) was utilized to inhibit the production of endogenous HIF1α, followed by Western blotting to analyze the degradation rates of HIF1α WT and the HIF1α R463K mutant. B. Statistical analysis corresponding to the data shown in (**B**); *n* = 3. **C** Western blot and immunoblot assays were conducted to assess the interaction potential between HIF1α and various autophagy-related proteins. **D** CHX treatment was applied to suppress endogenous HIF1α synthesis, and Western blotting was then performed to evaluate the degradation rates of HIF1α in TAX1BP1 WT and TAX1BP1 knockout (KO) cancer cells. **E** Statistical analysis of the results from (**D**). **F** Western blot and immunoblot assays were executed to examine the binding of HIF1α and TAX1BP1 in shNC and shPRMT6 cancer cells. **G** Western blot and immunoblot assessments were performed to determine the binding interactions of either HIF1α WT or the HIF1α R463K mutant with TAX1BP1. **H** Western blot analysis was carried out to evaluate the expression levels of HIF1α in various pre-treated cancer cell lines. Student’s t-test was applied for statistical evaluation. All Western blots were conducted in triplicate.

To further explore the relationship between HIF1α methylation and its autophagic degradation, we analyzed the degradation rates of HIF1α WT and the HIF1α R463 mutant. The results indicated that the HIF1α R463 mutant had a higher degradation rate compared to HIF1α WT (Fig. 6A, B). Given that cargo receptors are crucial for targeting proteins for autophagic degradation, we investigated which cargo receptor is necessary for HIF1α autophagic degradation. Western blot analysis revealed that HIF1α primarily interacts with TAX1BP1 (Fig. 6C). We then knocked out TAX1BP1 in LoVo cancer cells (Fig. S4G), and observed that TAX1BP1 knockout significantly inhibited HIF1α degradation (Fig. 6D). Furthermore, PRMT6 knockdown increased the interaction between HIF1α and TAX1BP1 (Fig. 6F). Consistently, overexpression of PRMT6 WT, but not the PRMT6 KLA mutant, decreased the binding of HIF1α to TAX1BP1 (Fig. S4H). The HIF1α R463K mutant also enhanced the binding between HIF1α and TAX1BP1 (Fig. 6G). Western blot analysis confirmed that TAX1BP1 knockout prevented the decrease in HIF1α levels caused by shPRMT6 (Fig. 6H). Overall, these data suggest that PRMT6-mediated asymmetrical di-methylation of HIF1α at arginine 463 enhances HIF1α expression by inhibiting its interaction with the autophagy-associated cargo receptor TAX1BP1.

### PRMT6-mediated tumor angiogenesis relied on HIF1α R463 asymmetrical di-methylation

**Fig. 7:**
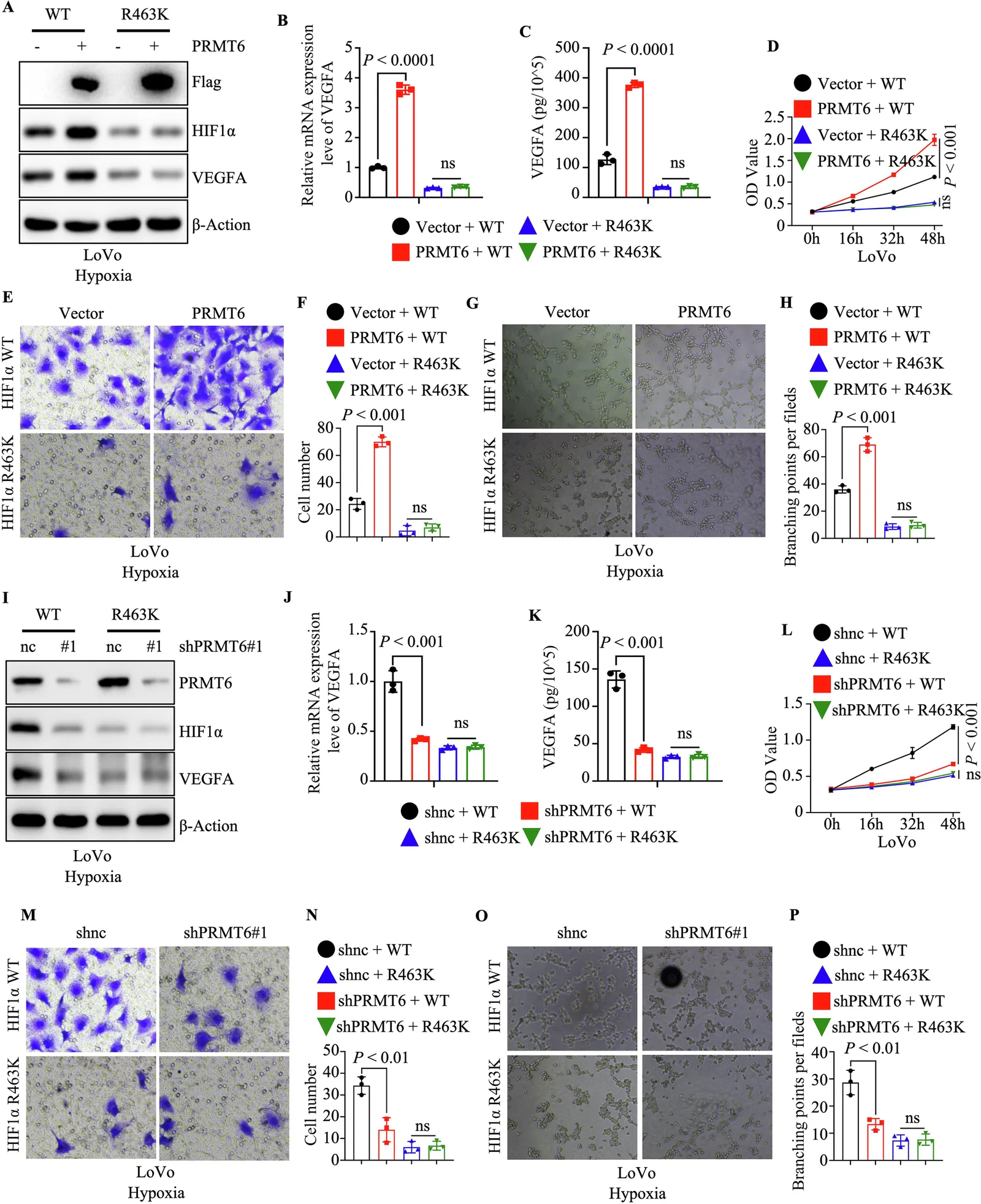
HIF1α R463 ADMA is crucial for PRMT6-mediated tumor angiogenesis in vitro. **A**, **I** Western blotting was conducted to assess the levels of targeted proteins in various pre-treated cancer cell lines. **B**, **C**, **J**, **K** RT-PCR and ELISA assays were performed to quantify VEGFA expression in the different pre-treated cancer cells; *n* = 3. **D**, **L**. CCK8 assays were used to evaluate the proliferation of HUVECs cultured with CM derived from various pre-treated cancer cells; *n* = 3. **E**, **M** Transwell assays assessed the migration capabilities of HUVECs exposed to CMs from the different pre-treated cancer cells. **F**, **N** Statistical analysis corresponding to (**E**, **M**) was included; *n* = 3. **G**, **O** Tube formation assays evaluated the ability of HUVECs to form tubular structures when cultured with CMs from various pre-treated cancer cells. **H**, **P** Statistical analysis for (**G**, **O**) was presented; *n* = 3. Student’s t-test or two-way ANOVA analysis was utilized for statistical analysis. All Western blots were executed in triplicate.

To further explore the connection between PRMT6 and HIF1α R463 ADMA in tumor angiogenesis, we established LoVo cancer cell lines that stably expressed various combinations: Vector + HIF1α WT, PRMT6 + HIF1α WT, Vector + HIF1α R463K, and PRMT6 + HIF1α R463K. Western blot analysis demonstrated that HIF1α R463K cells had reduced levels of both HIF1α and VEGFA compared to HIF1α WT cells. Additionally, PRMT6 overexpression elevated HIF1α and VEGFA levels in HIF1α WT cells, but this effect was absent in HIF1α R463K cells (Fig. 7A). This observation was further validated by RT-PCR and ELISA assays (Fig. 7B, C). In functional assays, HUVECs exposed to CM from HIF1α R463K cells exhibited decreased proliferation, migration, and tube formation capabilities compared to those treated with CM from HIF1α WT cells (Fig. 7D–H). Moreover, CM from PRMT6 + HIF1α WT cells significantly enhanced HUVEC proliferation, migration, and tube formation when compared to CM from Vector + HIF1α WT cells. In contrast, the effects were abolished in the presence of the HIF1α R463K mutant (Fig. 7D–H).

Next, we established LoVo cancer cell lines stably expressing shnc + HIF1α WT, shPRMT6 + HIF1α WT, shnc + HIF1α R463K, and shPRMT6 + HIF1α R463K. Western blot, RT-PCR, and ELISA assays confirmed that PRMT6 knockdown significantly reduced VEGFA expression in HIF1α WT cells but had no effect on HIF1α R463K cells (Fig. 7I–K). In functional assays, CM from shPRMT6 + HIF1α WT cells inhibited HUVEC proliferation, migration, and tube formation when compared to CM derived from shnc + HIF1α WT cells. However, these effects were abolished by the HIF1α R463K mutant (Fig. 7L–P). Collectively, these data demonstrate that HIF1α R463 asymmetrical di-methylation (ADMA) is crucial for PRMT6-mediated tumor angiogenesis in vitro.

### Targeting PRMT6 inhibited tumor angiogenesis in vivo

**Fig. 8:**
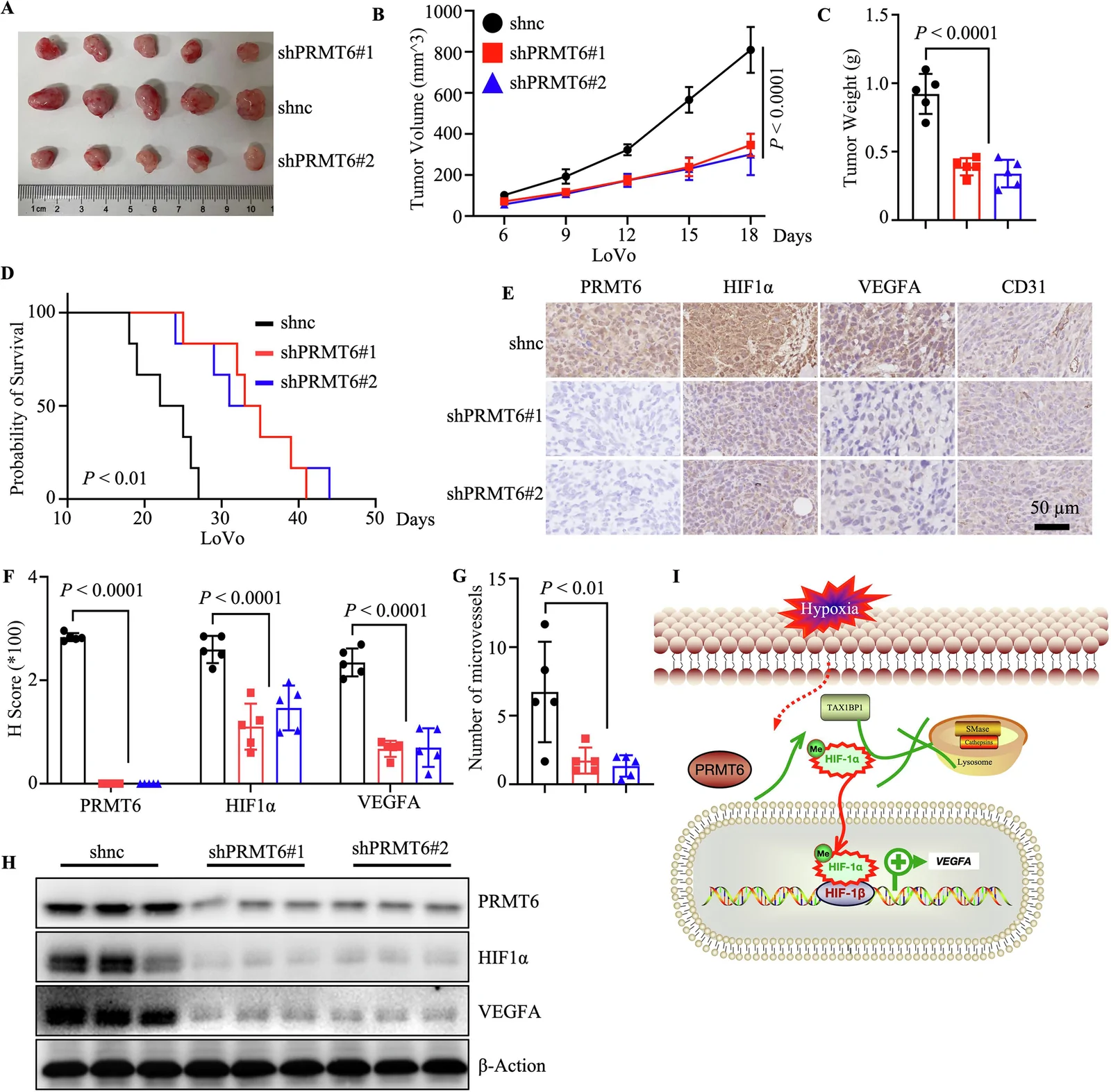
Knockdown of PRMT6 significantly inhibited tumor growth and angiogenesis in vivo. **A** Images showcasing the tumors; *n* = 5. **B** Graph depicting the tumor growth over time; *n* = 5. **C** Statistical evaluation of tumor weight; *n* = 5. **D** Kaplan-Meier survival analysis of mice; *n* = 6. **E** Representative images of IHC assays; *n* = 5; scale bar = 50 µm. **F** Quantitative analysis of IHC staining. The expression levels of PRMT6 and HIF-1α were evaluated using the H-score method; *n* = 5. **G** Statistical analysis of the number of intratumoral microvessels; *n* = 5. **H** Western blot analysis to assess the expression of targeted proteins in different tumors. **I** Working model: Hypoxia induced the binding of PRMT6 and HIF1α; PRMT6 asymmetrically di-methylated HIF1α at arginine 463 site, which suppressed the interaction of autophagy-related protein TAX1BP1 and HIF1α, and subsequently enhanced angiogenesis in colorectal cancer. Statistical analyses were conducted using Student’s t-test or one-way ANOVA analysis, and all Western blot experiments were executed in triplicate.

We then assessed the biological role of PRMT6 in vivo by injecting either shnc or shPRMT6 cancer cells into the backs of 8-week-old female nude mice. Tumor growth was monitored by measuring the major and minor axes every three days over a span of six days. After 18 days, the mice were euthanized, and tumors were harvested. Representative tumor images are displayed in Fig. 8A. Statistical analysis indicated that knockdown of PRMT6 significantly reduced both the tumor growth rate and weight (Fig. 8B, C). Survival analysis showed that PRMT6 knockdown significantly prolonged the overall survival of the mice (Fig. 8D). Immunohistochemistry (IHC) assays performed on the tumors revealed that silencing PRMT6 led to a significant decrease in HIF1α and VEGFA expression (Fig. 8E, F). Furthermore, using a CD31 antibody to identify microvessels, we found that PRMT6 knockdown markedly inhibited the formation of intratumoral microvessels (Fig. 8E, G). Western blot analysis of the tumor tissue proteins confirmed that PRMT6 knockdown reduced the expression levels of HIF1α and VEGFA (Fig. 8H). Collectively, these results suggest that PRMT6 may represent a promising target for anti-angiogenic therapy in colorectal cancer.

## Discussion

Given that the tumor microenvironment is frequently hypoxic, HIF1α emerges as a key regulator of tumor angiogenesis [19]. Enhancing the degradation of HIF1α within tumors could be an effective strategy to inhibit tumor progression. While PHD/VHL-mediated ubiquitination is the predominant degradation pathway for HIF1α, this process typically occurs under normoxic conditions, making it less ideal as a therapeutic target in hypoxic tumors [8, 20]. Our study highlights the importance of the autophagy-lysosome pathway in regulating HIF1α degradation. Although previous research has established that HIF1α can be degraded through autophagy, the specific mechanisms remain incompletely understood. We demonstrated that HIF1α is degraded via the autophagy-lysosome pathway, with the cargo receptor TAX1BP1 playing a critical role. These results broaden our insight into HIF1 signaling in tumor angiogenesis and indicate that facilitating the autophagic degradation of HIF1α could be a viable strategy for impeding tumor angiogenesis.

While previous studies, including our own, have established PRMT6 as an oncogenic driver in colorectal cancer (CRC) through mechanisms such as MYC signaling activation and epigenetic regulation of cell cycle-related genes [18]. However, the biological role of PRMT6 in tumor angiogenesis is still unclear. Here, this study unveils a novel and distinct function of PRMT6 in promoting CRC angiogenesis by stabilizing HIF-1α through asymmetric di-methylation at arginine 463 (R463 ADMA), thereby disrupting its interaction with the autophagy receptor TAX1BP1 and inhibiting lysosomal degradation. Importantly, this work represents the first demonstration that PRMT6 directly regulates HIF-1α stability via post-translational methylation, a mechanism distinct from its previously reported roles in transcriptional regulation or MYC pathway activation. The identification of PRMT6 as a pro-angiogenic factor expands its functional repertoire beyond tumor cell proliferation and survival, highlighting its dual oncogenic impact in CRC. Furthermore, our findings reveal that PRMT6-mediated HIF-1α stabilization drives VEGFA expression and vascularization, a pathway not previously linked to PRMT6 in any cancer type. The in vivo validation of PRMT6 knockdown significantly suppressing tumor angiogenesis and growth underscores its therapeutic potential as a target for anti-angiogenic strategies. In support of our genetic findings, we observed that the PRMT6 inhibitors SGC6870 and EPZ020411 effectively blocked HIF-1α methylation in vitro. These results suggested that the PRMT6–HIF-1α axis was pharmacologically targetable, providing a strong rationale for the future development of PRMT6-based anti-angiogenic therapies in CRC patients.

Notably, our results suggest that PRMT6 promotes angiogenesis even under normoxia. While we identified the PRMT6-HIF-1α axis as a key driver, HIF-1α–independent mechanisms may also exist. As a methyltransferase, PRMT6 might directly activate the transcription of pro-angiogenic factors via H3R2 methylation, or modulate non-histone targets like NF-κB or STAT3. Such parallel pathways could ensure robust vascularization across varying oxygen gradients, a possibility that warrants further methylome-wide investigation. Besides, VEGFA served as a key representative factor in our study to demonstrate the activation of the PRMT6–HIF-1α signaling axis. Given that HIF-1α was a master regulator of numerous angiogenic factors, it was highly likely that other downstream targets also participated in PRMT6-driven tumor vascularization. Future secretome-wide analyses would further elucidate the complete landscape of PRMT6-regulated cytokines in the CRC microenvironment. Beyond the direct regulation of angiogenic signaling by CRC cells, the tumor microenvironment—including infiltrating inflammatory cells and cancer-associated fibroblasts—also significantly influenced vascularization. Although our study focused on the PRMT6–HIF-1α–VEGFA axis, the potential impact of PRMT6 on other stromal compartments in CRC remained to be further elucidated.

In conclusion, our study demonstrated that PRMT6 promotes tumor angiogenesis by asymmetrically methylating HIF1α at the arginine 463 site. This modification, HIF1α R463 ADMA, inhibits the autophagy-lysosome pathway degradation of HIF1α by disrupting its interaction with the cargo receptor TAX1BP1 (Fig. 8I). These findings suggest that PRMT6 may be an effective target for therapeutic strategies aimed at inhibiting colorectal tumor angiogenesis.

## Methods and material

### Cell lines

The LoVo, HCT116, HEK293T, and HUVEC cell lines were sourced from the American Type Culture Collection (ATCC, Manassas, VA, USA). LoVo cells were cultivated in F-12K Medium (21127030, Gibco). HCT116 cells were kept in McCoy’s 5 A Medium (16600082, Gibco). HEK293T cells were cultivated in DMEM Medium (11965092, Gibco). All cell lines were maintained at 37 °C in a controlled atmosphere of 95% air and 5% CO_2_. For hypoxia treatment, cells were cultured in a hypoxia incubator maintained at 1% O_2_, 5% CO_2_, and 94% N_2_ at 37 °C for 8 h. The cell line was recently authenticated using Short Tandem Repeat (STR) profiling and was confirmed to be free of mycoplasma contamination.

### Cell lines construction

For gene silencing, shRNA sequences were cloned into pLKO.1-shRNA. For overexpression, cDNA was cloned into pLKO-cDNA. Lentiviruses were packaged using psPAX2 and pMD2.G (4:3:1 ratio) in HEK293T cells. Target cells were infected and selected with 2 μg/mL puromycin for 7 days to establish stable cell lines. The mutant plasimds constructs were generated using the Mut Express II Fast Mutagenesis Kit V2 (Vazyme, C214) according to the manufacturer’s protocol. The knockout (KO) cell lines were generated using the CRISPR-Cas9 system. Single-cell clones were obtained by limiting dilution, and KO efficiency was validated by genomic DNA sequencing and Western blot. All shRNA and gRNA sequences were listed as followed: shPRMT6#1: 5’- GCGAACCAAGTGGCTGAAGGA -3’; shPRMT6#2: 5’- CACGGACGTTTCAGGAGAGAT -3’; shPRMT6#3: 5’- ACCAGTGGAGACTGTAGAGTT -3’; sgBeclin1#1: 5’- GCGTCTGGGCATAACGCATC -3’; sgBeclin1#2: 5’- CCTGGACCGTGTCACCATCC -3’; sgBeclin1#3: 5’- GGACACGAGTTTCAAGATCC -3’; sgATG5#1: 5’- TCCGATTGATGGCCCAAAAC -3’; sgATG5#2: 5’- TTCCATGAGTTTCCGATTGA -3’; sgATG5#3: 5’- ACCAGTTTTGGGCCATCAAT -3’;

### Agents and antibodies

Antibodies for HIF-1α (36169, CST), HIF-2α (A7553, Abclonal), PRMT6 (14641, CST), CD31 (ab28364, Abcam), VEGFA (ab52917, Abcam), β-Actin (4970, CST), Flag-tag (14793, CST), ATG5 (12994, CST), Beclin1 (3738, CST), Mono-Methyl Arginine (8711, CST), Asymmetric Di-Methyl Arginine (13522, CST), HA-tag (3724, CST), TAX1BP1 (5105, CST), HIF-1β (5537, CST), H3R2me2a (A3155, CST), HRP-conjugated Goat anti-Rabbit IgG (H + L) (AS014, Abclonal), and HRP-conjugated Goat anti-Mouse IgG (H + L) (AS003, Abclonal) were purchased from the specified companies. All primary antibodies were diluted in Universal Antibody Dilution Buffer (RM02955, Abclonal) or 5% non-fat milk. Cycloheximide (HY-12320), MG-132 (HY-13259), Chloroquine (HY-17589A), EPZ020411 (HY-12970), and SGC6870 (HY-126300) were obtained from MedChemExpress.

### Enzyme-Linked Immunosorbent Assay (ELISA)

The VEGFA level of conditioned medium (CM) was measured by Human VEGFA ELISA Kit (RK00023, ABclonal) according to the instruction book.

### Real-time Polymerase Chain Reaction (RT-PCR)

Total RNA was extracted using TRIzol (R0016, Beyotime) and subsequently converted to cDNA with the BeyoRT™ III First Strand cDNA Synthesis Master Mix (5X) (including gDNA EZeraser) (D7185M, Beyotime). Reverse transcription PCR (RT-PCR) was conducted with BeyoFast™ SYBR Green qPCR Mix (2X, Low ROX) (D7262, Beyotime). The primers used for VEGFA and HIF1α were as follows: VEGFA Forward: 5’-AGGGCAGAATCATCACGAAGT-3’, Reverse: 5’-AGGGTCTCGATTGGATGGCA-3’; HIF1α Forward: 5’-GAACGTCGAAAAGAAAAGTCTCG-3’, Reverse: 5’-CCTTATCAAGATGCGAACTCACA-3’.

### CCK8 assays

The proliferative capacity of pre-treated HUVECs was assessed using the Cell Counting Kit-8 (C0037, Beyotime) following the manufacturer’s instructions.

### Trans-well assays

HUVECs, numbering between 40,000 and 100,000, were suspended in 100 µl of serum-free medium and added to the upper chamber (3464, Corning). The lower chamber was filled with CMs to induce migration. The trans-well plate was incubated at 37 °C in a CO_2_ incubator for about 12–16 h. After the incubation period, non-migrated cells in the upper chamber were carefully wiped away using a cotton swab. The cells that had migrated to the lower surface of the membrane were fixed with 4% paraformaldehyde and stained with 1% crystal violet. The stained cells were then observed and counted under a microscope to determine their migration ability. For quantification, five random fields per chamber were selected to count the migrated cells, and the average value was used for statistical analysis. All experiments were performed in triplicate.

### Tube formation assays

To evaluate the angiogenic capacity of HUVECs, a tube formation assay was performed using Matrigel (CLS354230, Sigma). Briefly, pre-chilled 96-well plates were coated with 100 µL of growth factor-reduced Matrigel per well and incubated at 37 °C for 30–60 min to allow for polymerization. HUVECs were then seeded onto the Matrigel at a density of 2–3 × 10^4^ cells per well in 100 µL of CMs. The plate was incubated in a 5% CO_2_ atmosphere at 37 °C. Capillary-like tube structures were monitored periodically using an inverted microscope. For quantification, representative images were captured at 6 h post-seeding, and the number of branching points, defined as junctions where three or more tube segments converged, was quantified using ImageJ software (version 1.53e). Statistical analysis was performed using the raw counts of branching points per field.

### Western blot

Cells were lysed with NP-40 lysis buffer containing protease and phosphatase inhibitors. Protein concentrations were determined using a BCA assay. The protein samples were combined with SDS sample buffer and heated at 100 °C for 5 min. Equal amounts of protein were then loaded into each well, and the SDS-PAGE gel was run at a steady voltage (commonly 80 V for the stacking gel and 120 V for the resolving gel). Following this, proteins were transferred to a PVDF membrane at 100 V for 2 h. To block non-specific binding, the membrane was treated with 5% non-fat milk in TBST for 1 h at room temperature. The next step involved overnight incubation at 4 °C with a primary antibody diluted in the blocking buffer (5% non-fat milk in TBST). The dilution factor for each antibody was strictly determined according to the manufacturer’s recommended instructions to ensure optimal signal specificity. The following day, the membrane was incubated for 1 hour at room temperature with an HRP-conjugated secondary antibody, also diluted in the blocking buffer (1:5000). The chemiluminescent signals were detected using an ECL substrate.

### Immunoprecipitation assays

In the immunoprecipitation assays, we used equal volumes of cell lysates derived from the same number of cells harvested from culture dishes of identical specifications. The protein supernatant was incubated with magnetic beads (HY-K0202, MCE) for 1–2 h to reduce non-specific interactions. The beads were separated by centrifugation or a magnetic rack, and the supernatant was collected. A specific antibody was added to the supernatant and incubated overnight at 4 °C with rotation. The antibodies were diluted according to the recommended concentrations provided in the manufacturers’ instructions. Afterward, magnetic beads were added and incubated at 4 °C with rotation for an additional 1–2 h. The beads were then washed 3–5 times with NP-40 lysis buffer, each wash lasting 5 min, to eliminate any non-specifically bound proteins. Finally, bound proteins were eluted from the beads by adding SDS sample buffer and heating at 95 °C for 5 min, followed by Western blot analysis.

### Immunohistochemistry (IHC)

A total of 69 pairs of matched colorectal tumor tissues and adjacent normal tissues were collected from patients who underwent surgical resection at Qilu Hospital, Shandong University. Tissue samples were preserved in formalin and subsequently embedded in paraffin. Sections with a thickness of 4–5 µm were prepared using a microtome, followed by deparaffinization, rehydration, antigen retrieval (Heat-induced epitope retrieval), blocking, antibody incubation, and imaging. All primary antibodies were diluted strictly according to the manufacturer’s recommended concentrations for IHC applications (typically ranging from 1:100 to 1:200). Secondary antibodies were used at a 1:500 dilution. Signal visualization was performed using the DAB substrate kit (ab64238, abcam). The reaction was monitored under a microscope and terminated with distilled water once the desired brown staining intensity was achieved. Sections were then counterstained with hematoxylin for 60 s, dehydrated through a graded ethanol series, cleared in xylene, and mounted with neutral resin. For each slide, five random, non-overlapping fields were captured. Images were acquired at 200× magnification for general assessment. Microvessel density (MVD) was quantified based on CD31-positive staining. Areas of highest vessel density were identified at low power, and then five random fields per sample were counted at 200× magnification. To account for intra-tumoral heterogeneity, three non-overlapping sections were obtained from different depths of subcutaneous tumor. Within each section, five random high-power fields (HPFs) were captured, and the number of microvessels was counted and averaged. The final data represent the sectional MVD measurements to reflect the overall angiogenic status.

The immunohistochemistry (IHC) results for clinical samples were assessed by two independent experts based on the following criteria: Intensity Scoring, 0: No staining, 1: weak staining, 2: moderate staining, 3: strong staining; Extent (Percentage of Positive Cells) Scoring, 0: 0% of cells stained, 1: 1–25% of cells stained, 2: 26–50% of cells stained, 3: 51–75% of cells stained, 4: 76–100% of cells stained. IHC score was calculated by the formula: Intensity Scoring multiplied Extent Scoring. High expression was defined as an IHC score > 7, while a score ≤ 7 indicated low expression.

The protein expression levels in subcutaneous xenograft tissues were quantified using the H-score (histological score) method. Staining intensity was graded on a four-point scale: 0 (no staining), 1+ (weakly positive), 2+ (moderately positive), and 3+ (strongly positive). The percentage of positive cells (0–100%) for each intensity level was recorded. The final H-score was calculated using the following formula: H-score = [1 × (% of 1 + )] + [2 × (% of 2 + )] + [3 × (% of 3 + )].

### Animal experiments

Six to eight-week-old female nude mice (D000521) were obtained from GemPharmatech. The mice were randomly assigned into three experimental groups using a random number table to ensure an unbiased distribution of animals. All mice were housed under identical environmental conditions throughout the study. LoVo cells in the logarithmic growth phase were harvested, and washed twice with ice-cold PBS. Pre-treated lovo cells (5 × 10^5, 100 μl of PBS) were subcutaneously injected into the right flank of the mice. Tumor growth at the implantation site was monitored, and tumor volume was recorded along with any morphological changes. Tumor volume was determined using the formula: 0.5 × (major axis) × (minor axis)². After 18 days, all mice were euthanized, and tumor tissues were collected for subsequent analysis. No animals or samples were excluded from the analysis. All experimental data were included for statistical evaluation.

### Statistical analysis

Bioinformatics analysis was conducted using the TCGA database, GEO database (https://www.ncbi.nlm.nih.gov), and GEPIA tool. Statistical analyses were performed using GSEA software (Version 4.3.3) and GraphPad Prism (Version 9.0). The normality of data distribution was assessed using the Shapiro-Wilk test. All data are presented as mean ± SD (standard deviation). Student’s t-test was used for two-group comparisons if variances were equal. If variances were unequal, the Mann-Whitney U test was performed. One-way or two-way ANOVA was used for multiple-group comparisons. Survival curves were estimated using the Kaplan-Meier method, and the significance of differences between groups was determined using the log-rank test. A *p*-value of less than 0.05 was considered indicative of statistical significance.

## Supplementary information

Supplementary figure legend

Supplementary figure 1

Supplementary figure 2

Supplementary figure 3

Supplementary figure 4

The full uncropped Gels and Blots image

## Data Availability

All data and materials used in the study were available in the manuscript.
