# Supplementary material for: PRMT6 acts as a pro-angiogenic factor in colorectal cancer
Source: Cell Death Discov. 2026 Jul 11;12:402. doi: 10.1038/s41420-026-03256-y (PMC13639044; doi:10.1038/s41420-026-03256-y)

Figure 1H

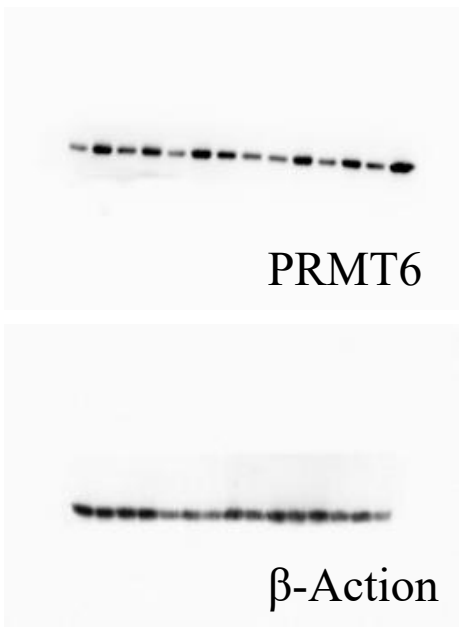

Figure 2A

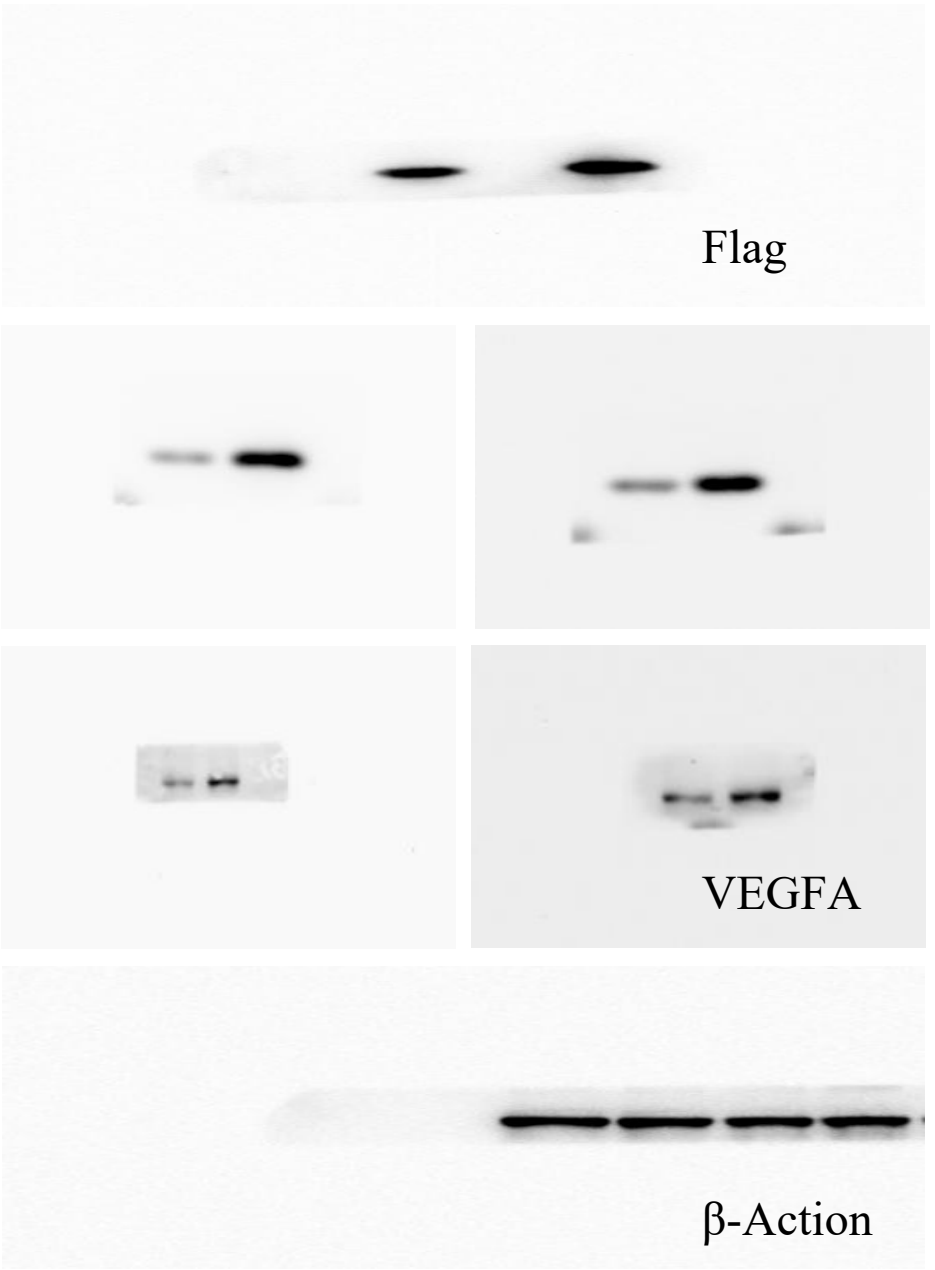

Figure 3A

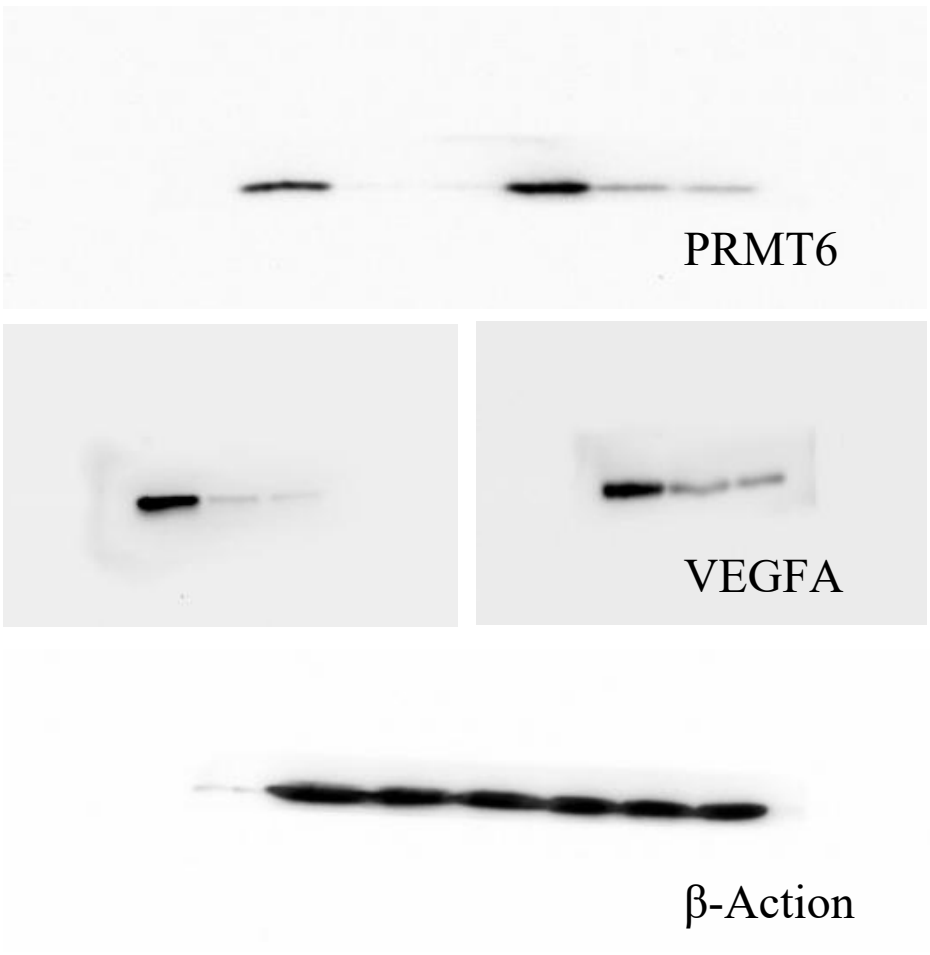

Figure 4B

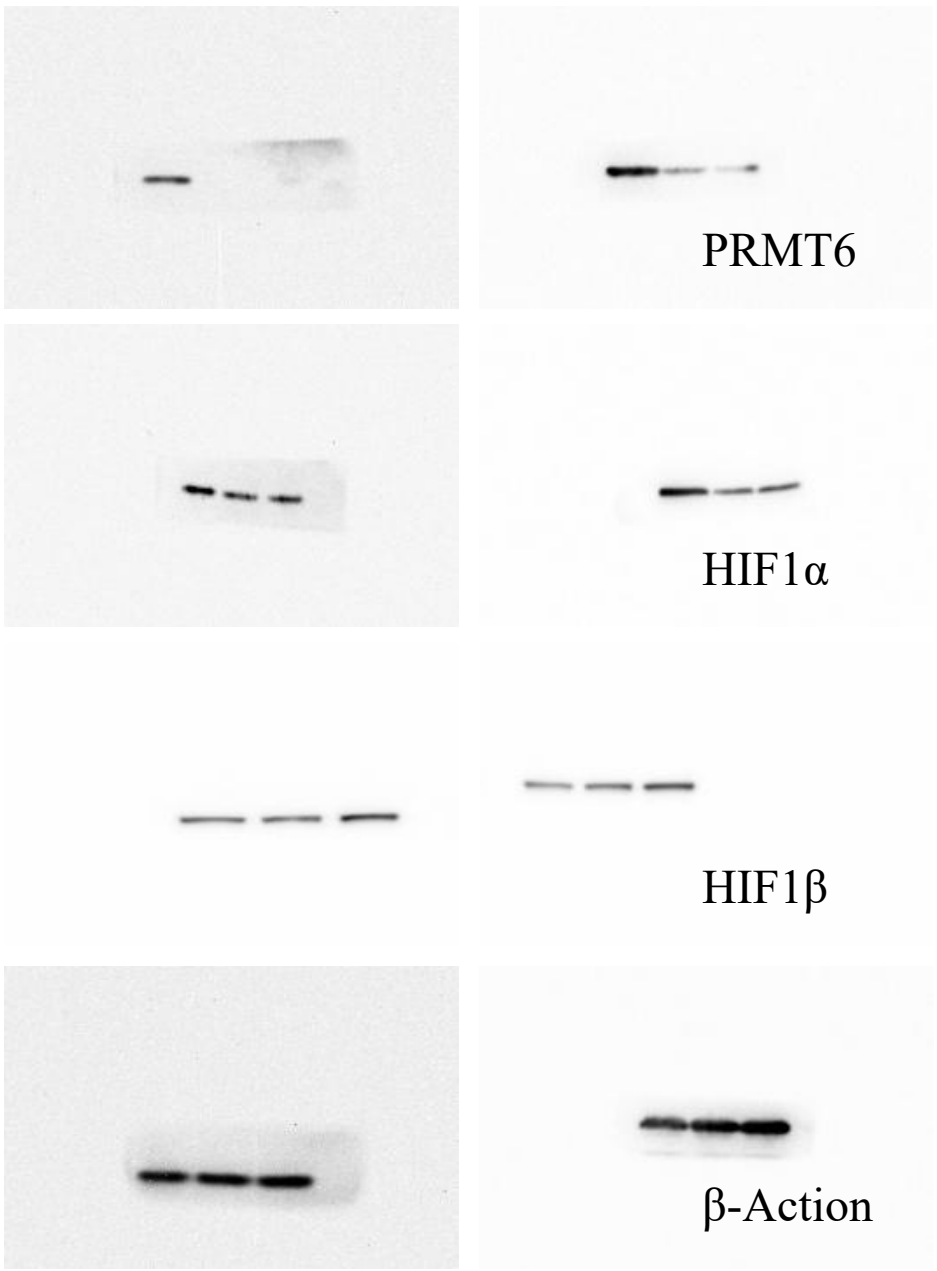

Figure 4C

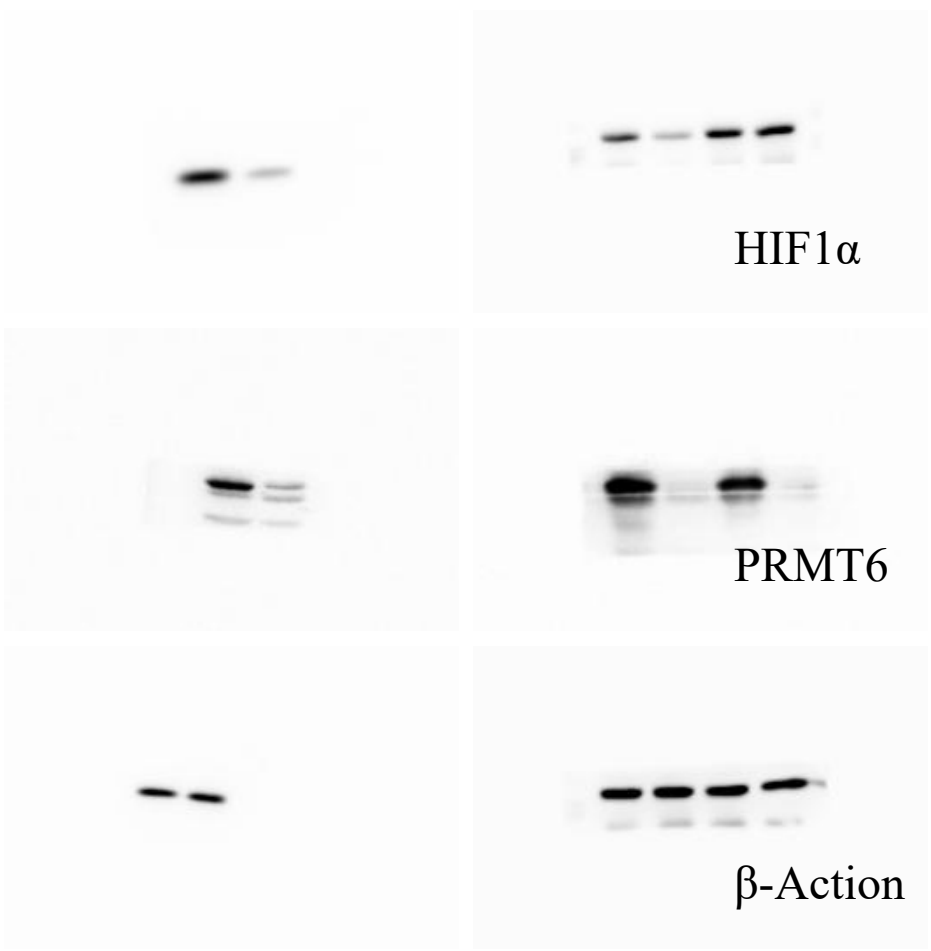

Figure 4D

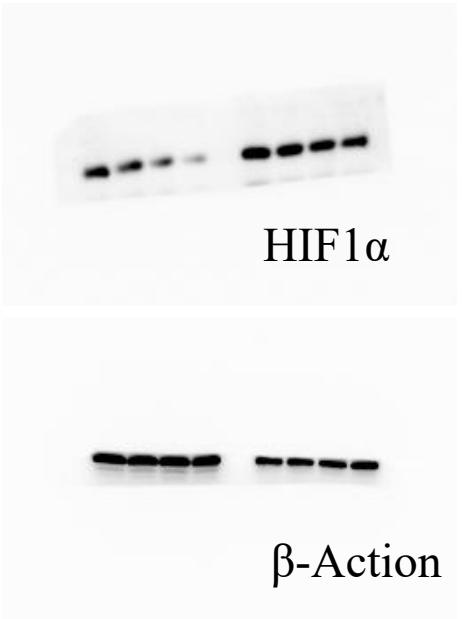

Figure 4F

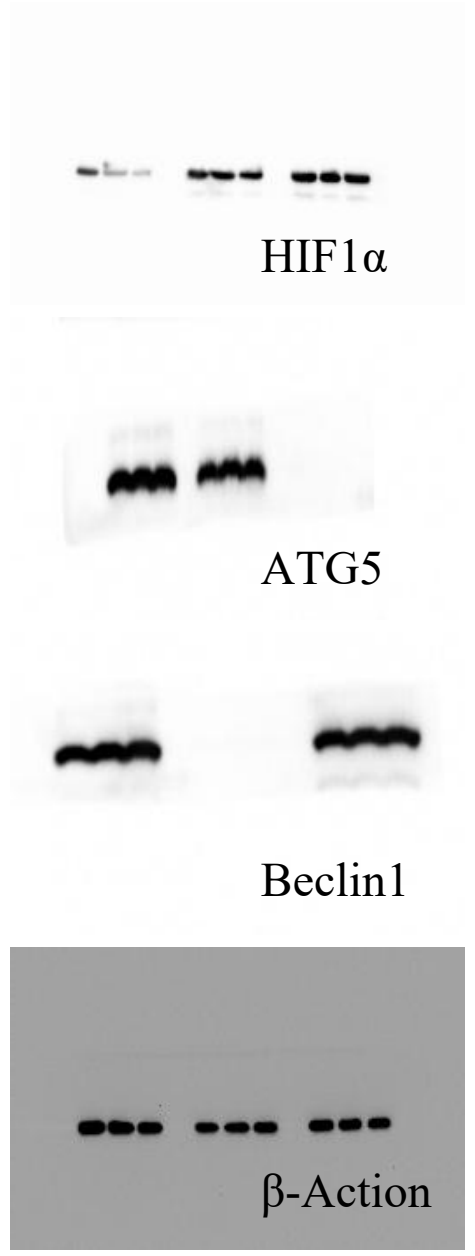

Figure 4H

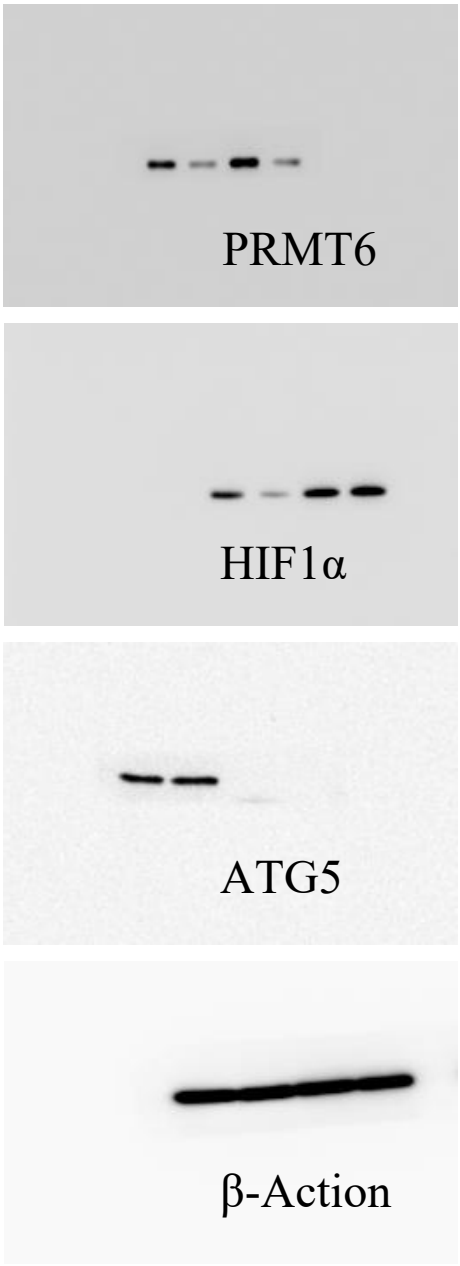

Figure 4I

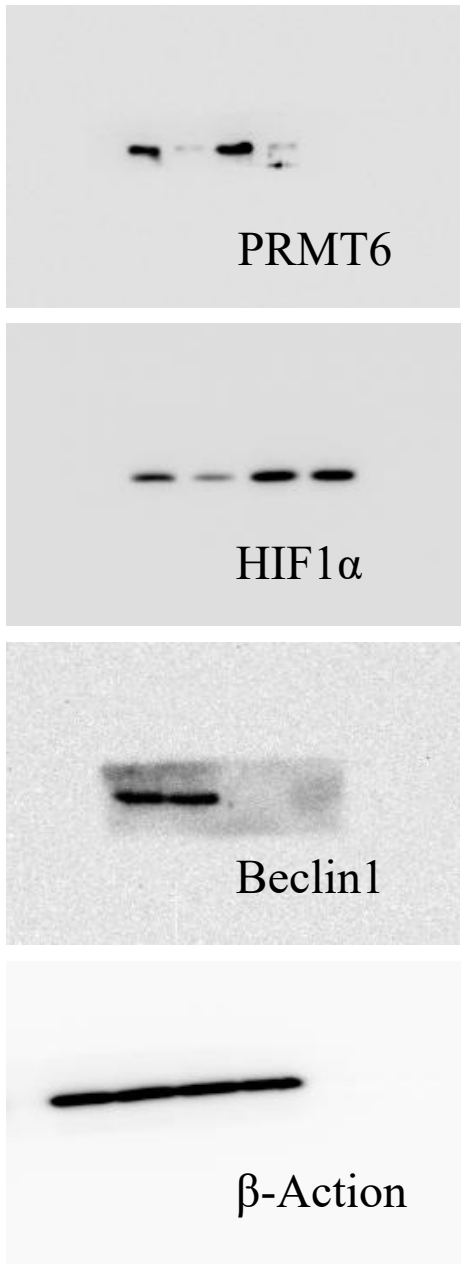

Figure S3B

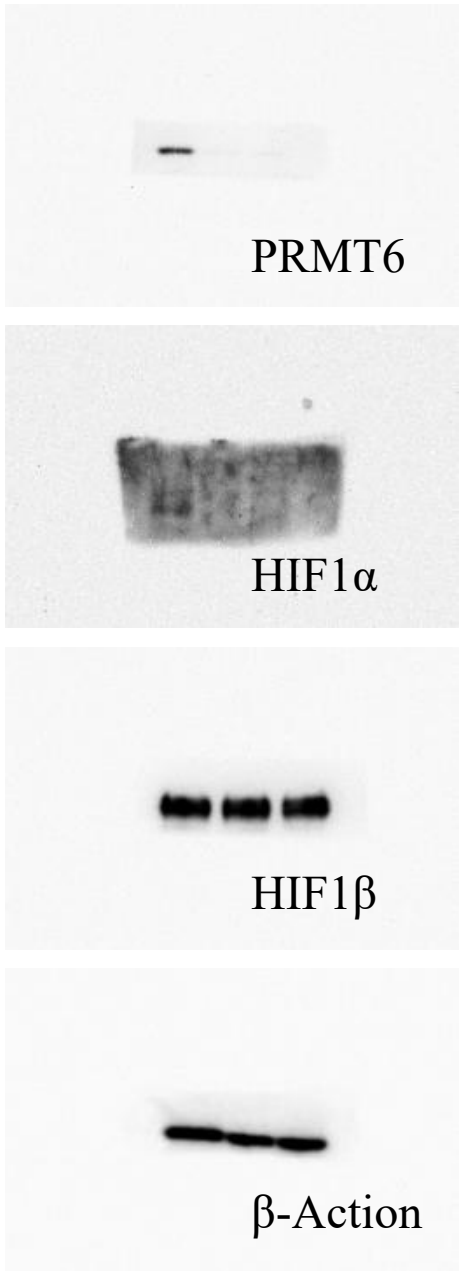

Figure S3C

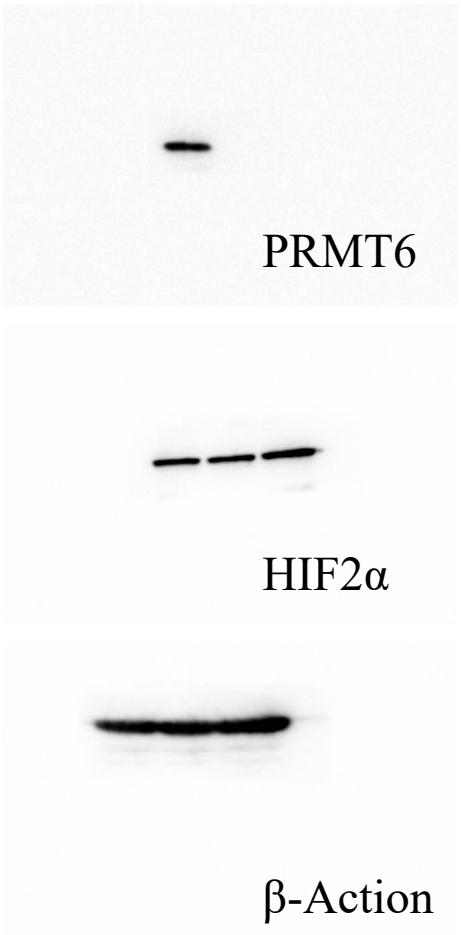

Figure S3D

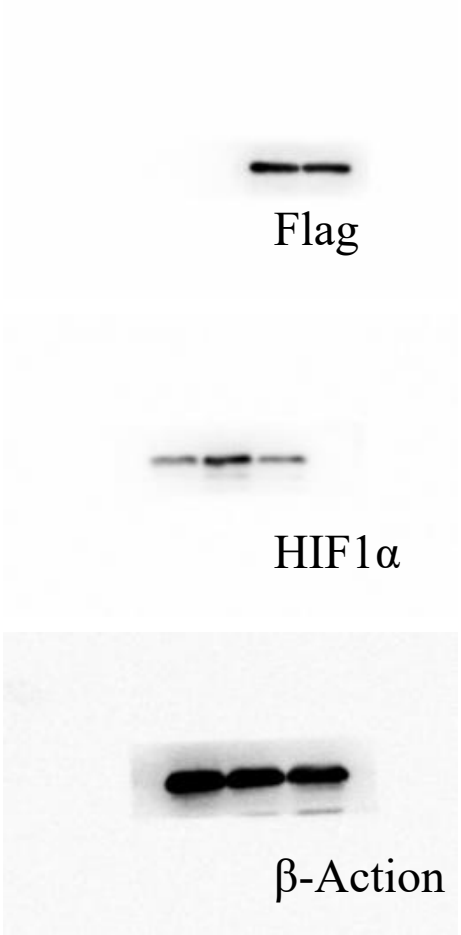

Figure S3E

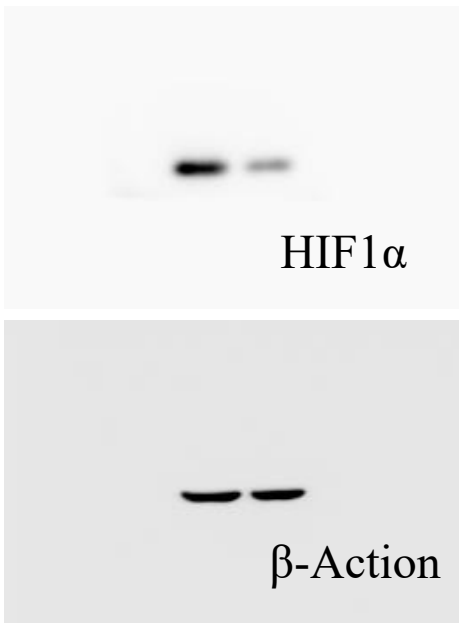

Figure 5A

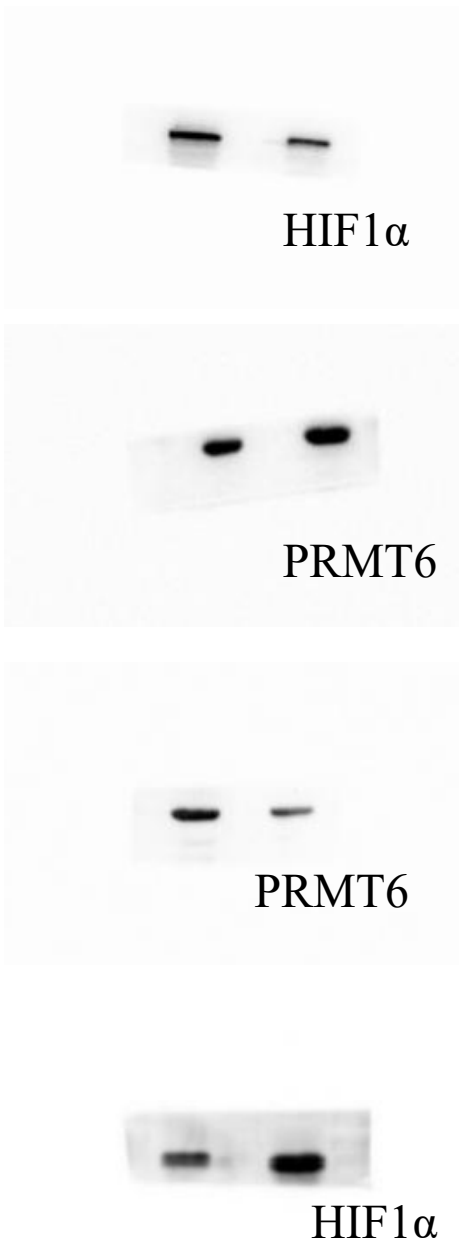

Figure 5B

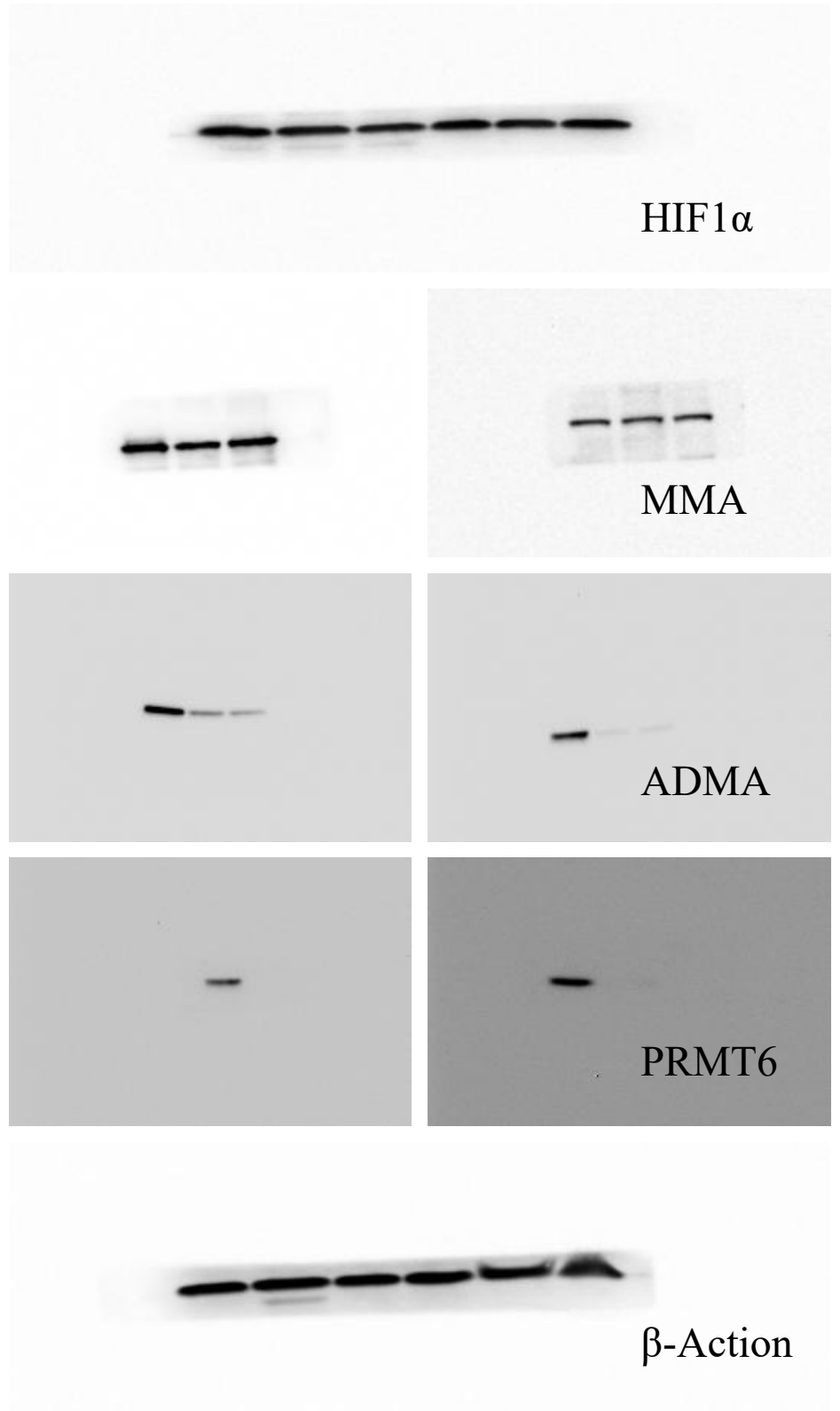

Figure 5C

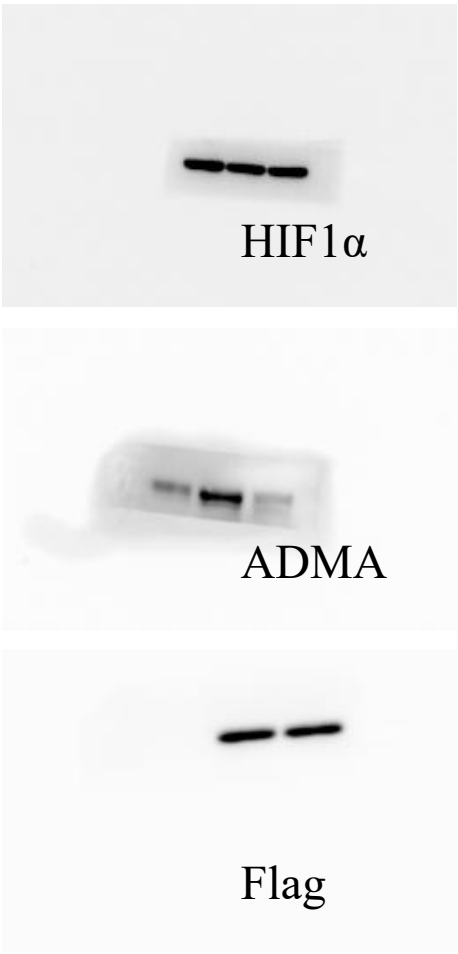

Figure 5D

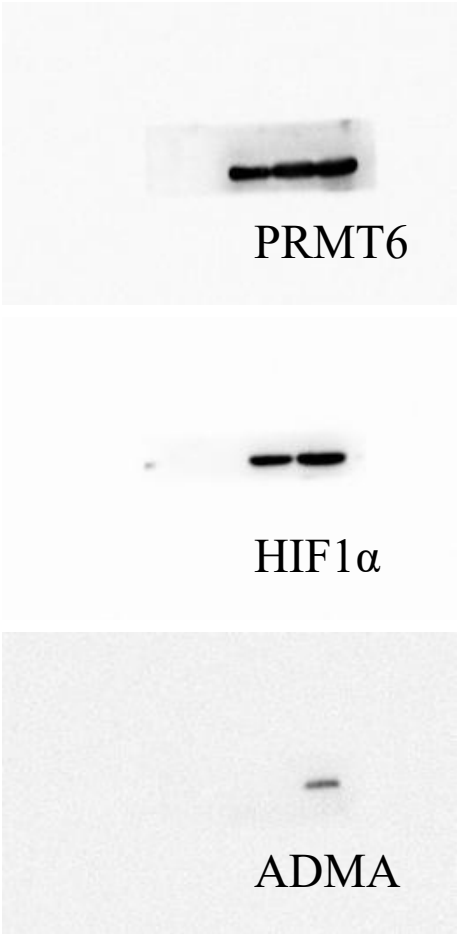

Figure 5F

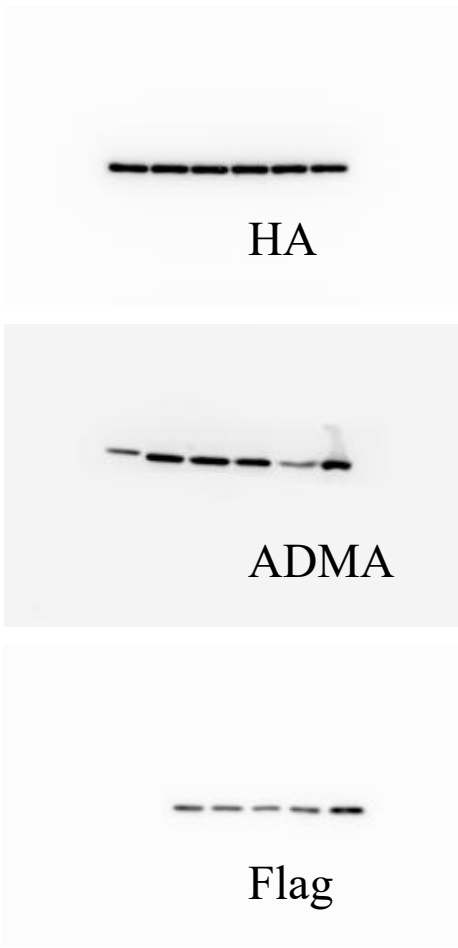

Figure 5G

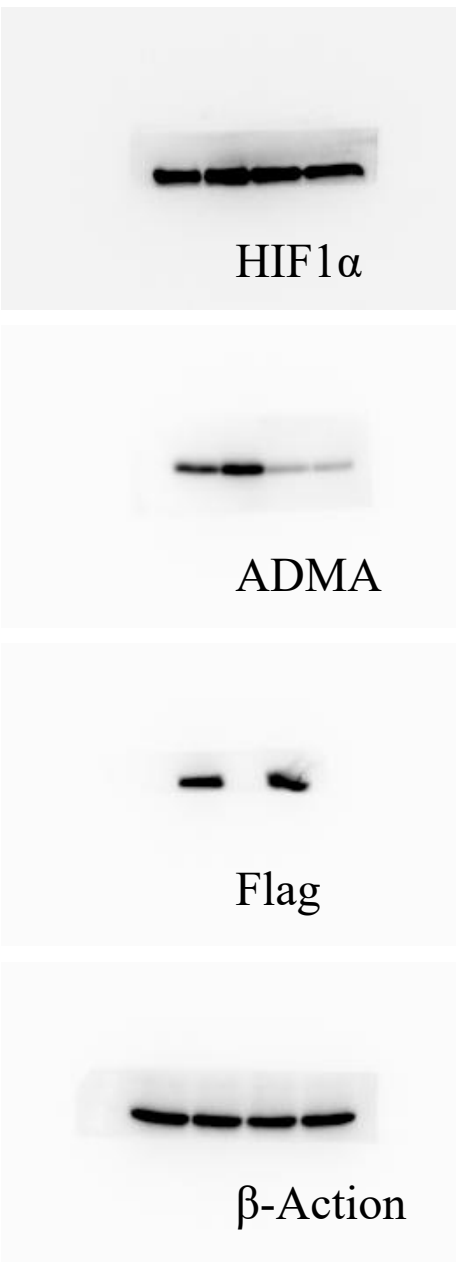

Figure 6A

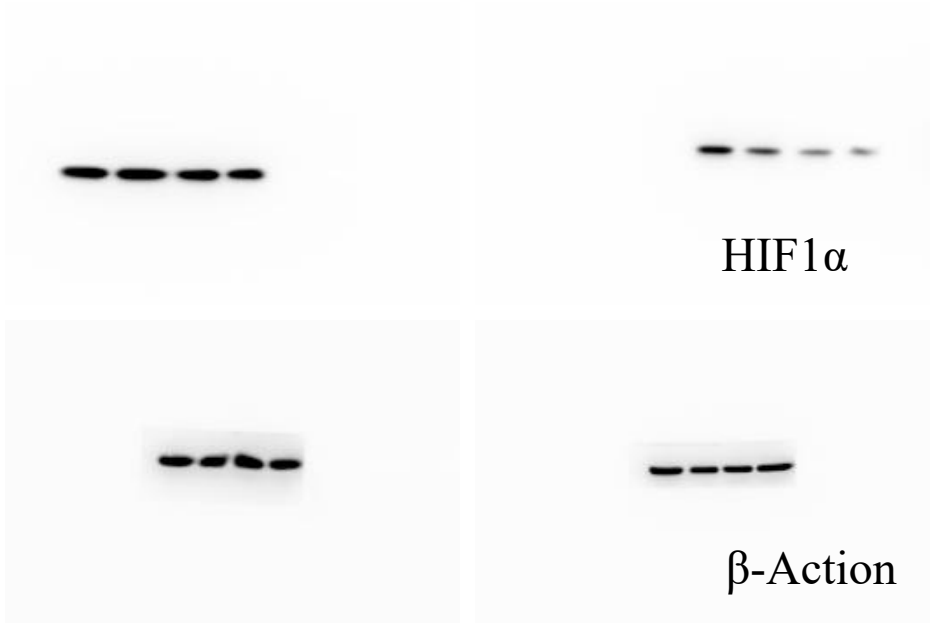

Figure 6C

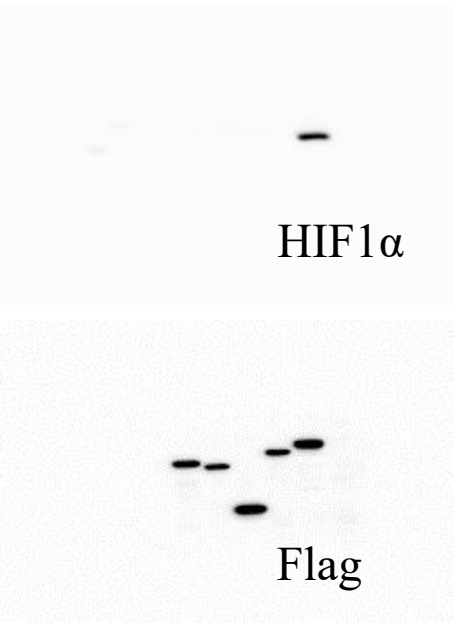

Figure 6D

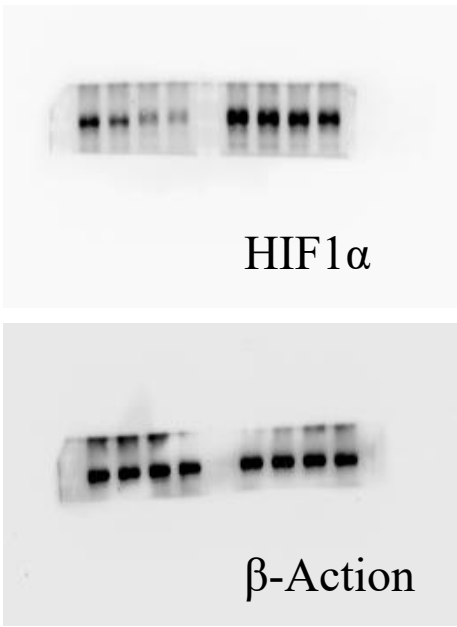

Figure 6F

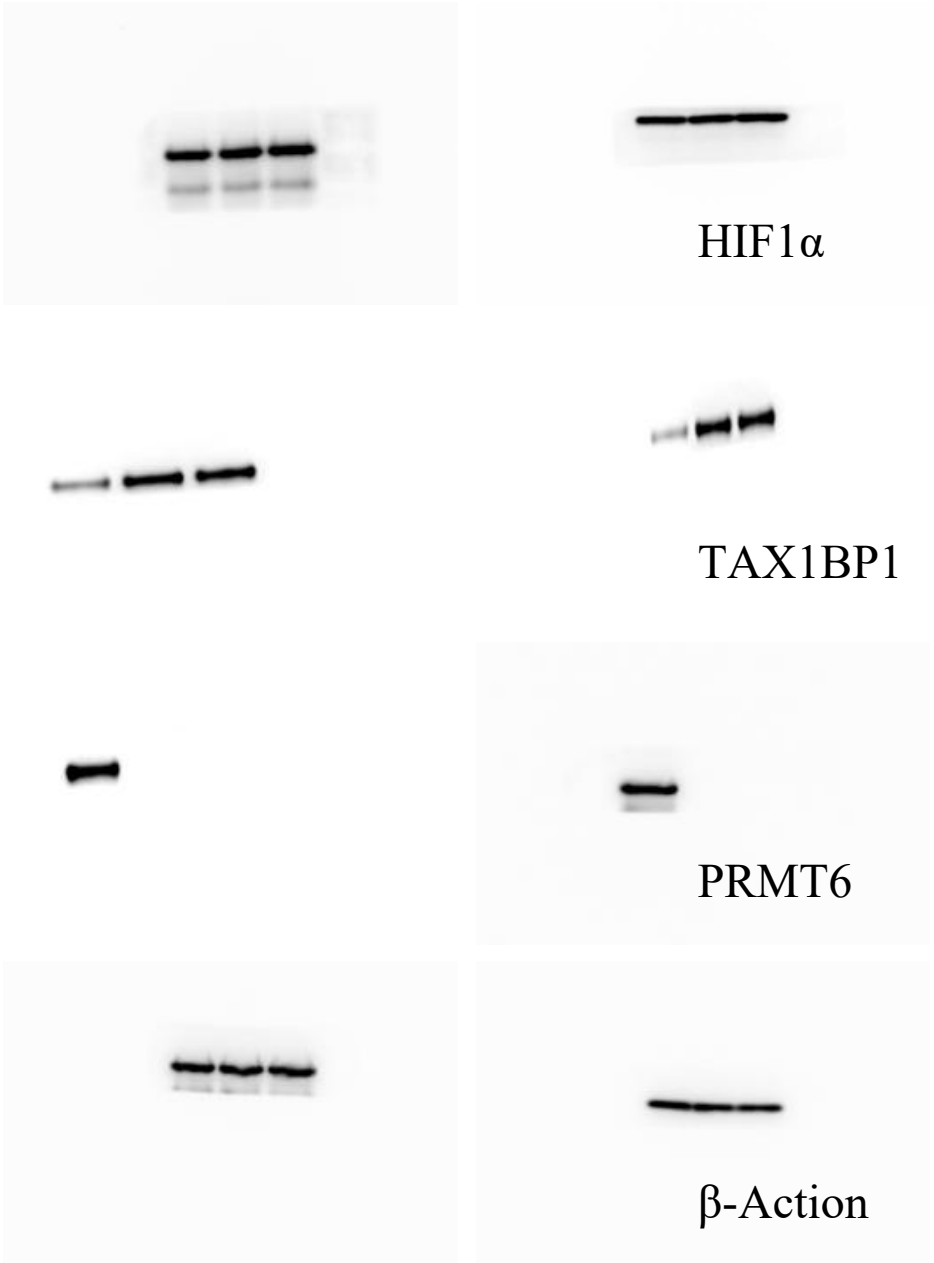

Figure 6G

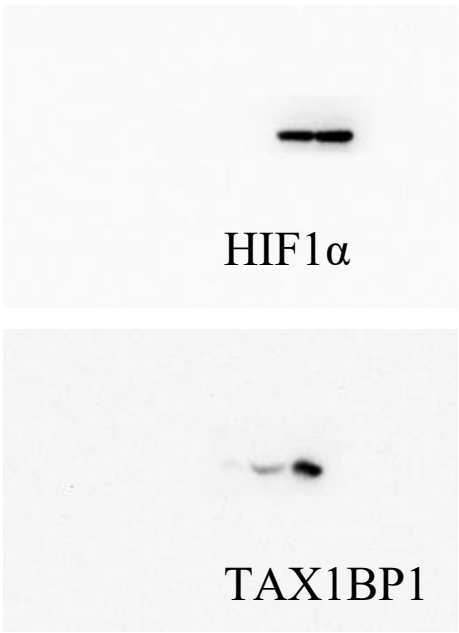

Figure 6H

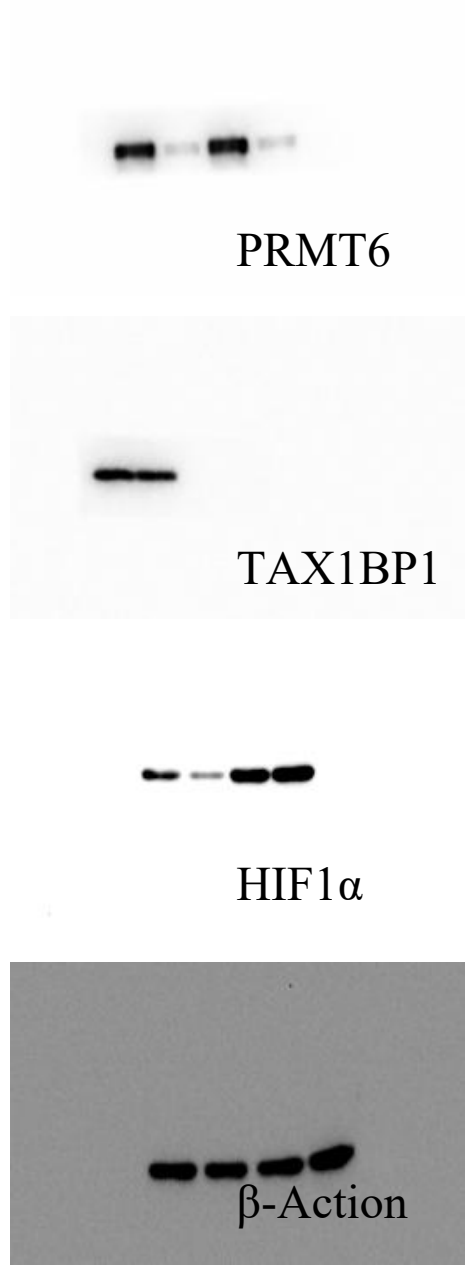

Figure S4B

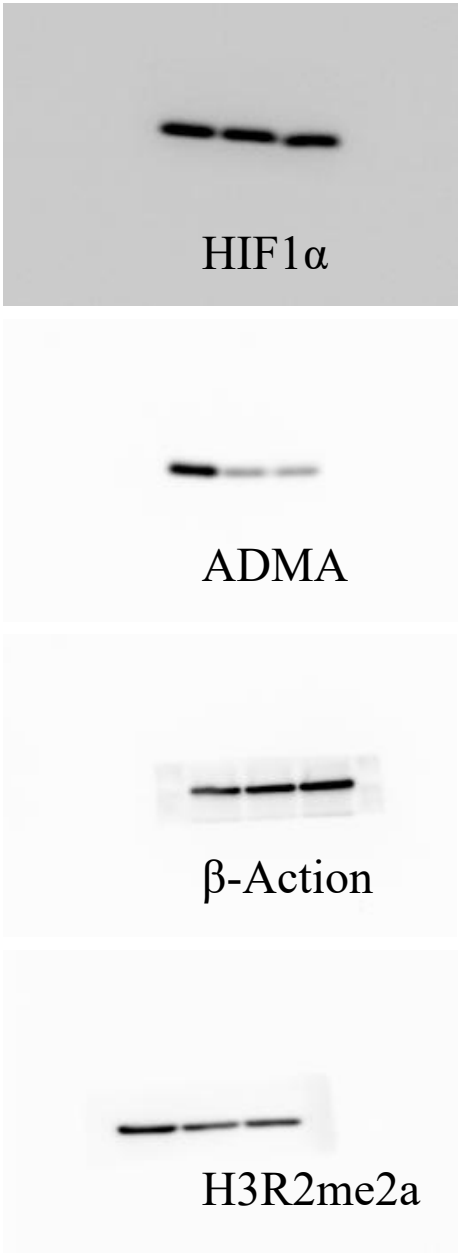

Figure S4C

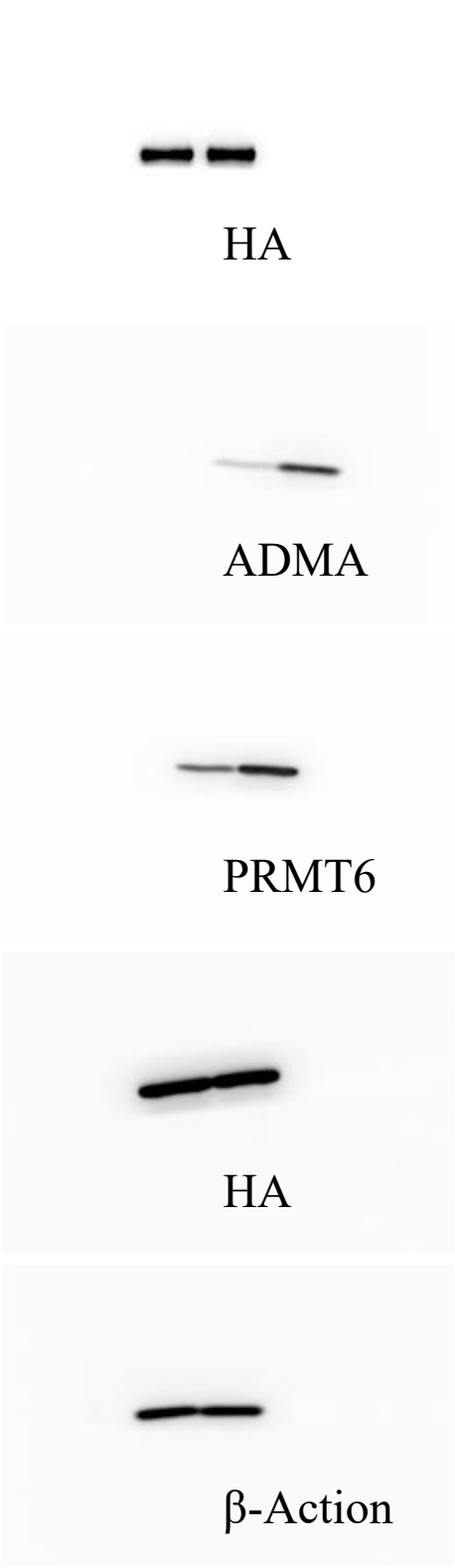

Figure S4D

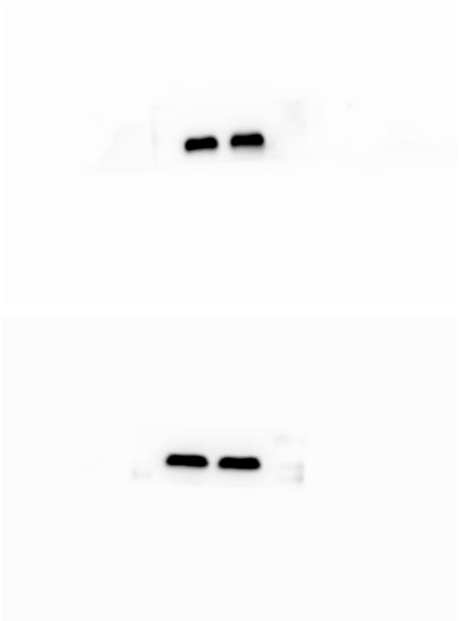

Figure S4E

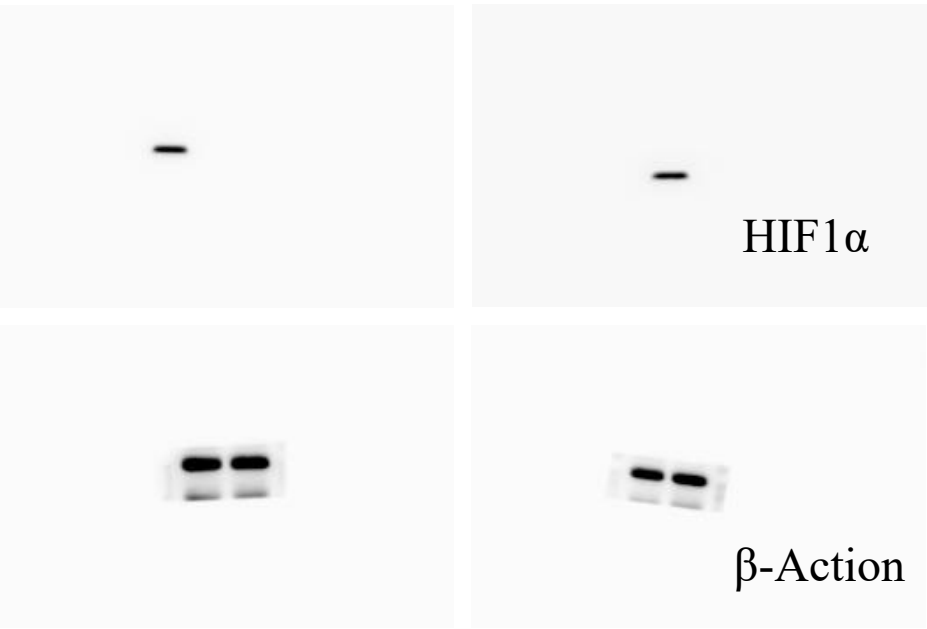

Figure S4F

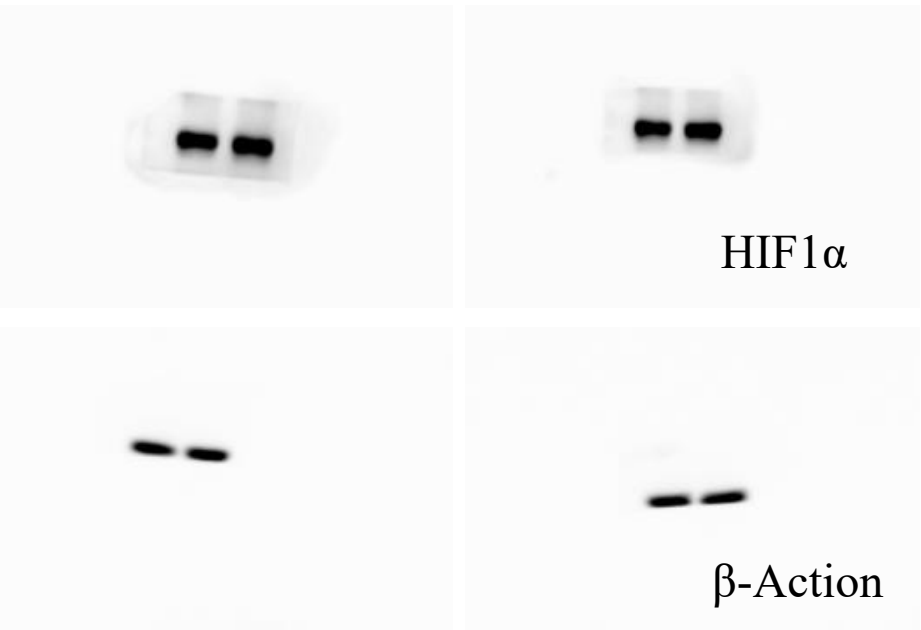

Figure S4G

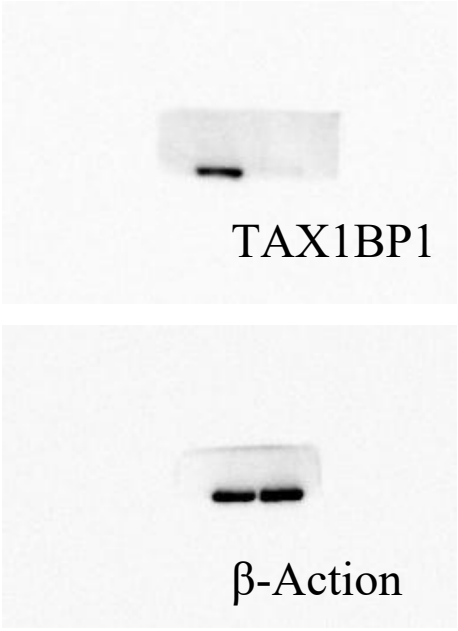

Figure S4H

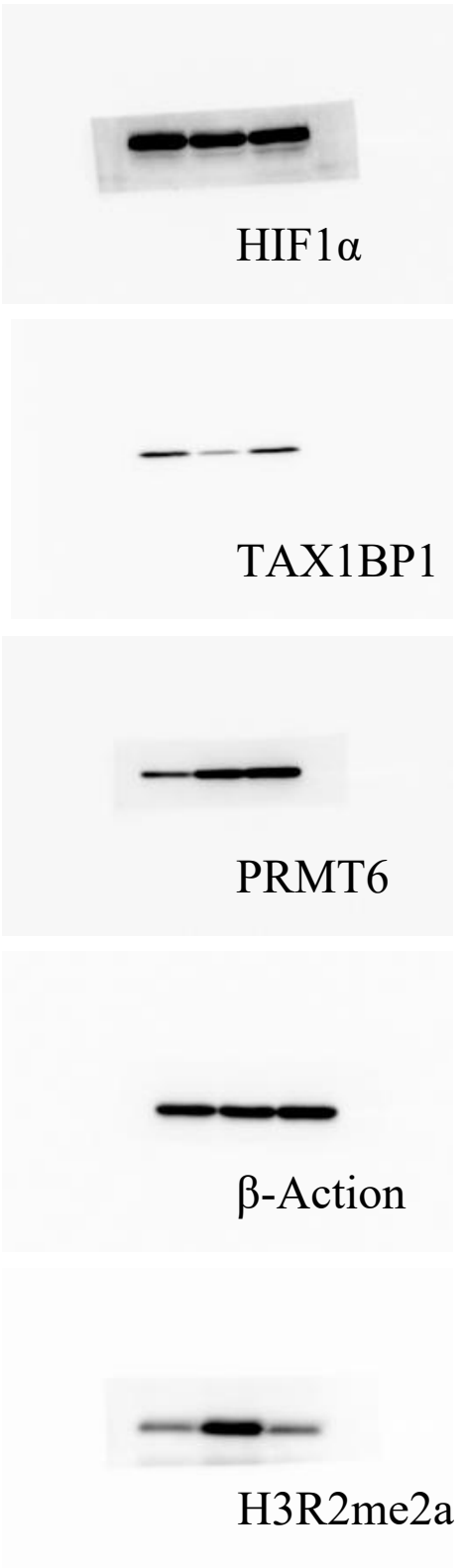

Figure 7A

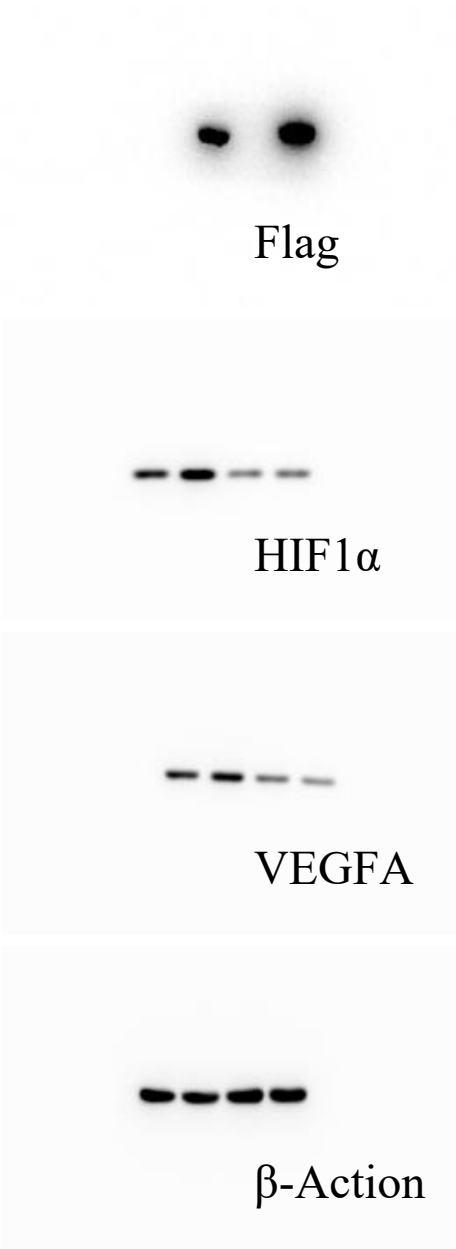

Figure 7I

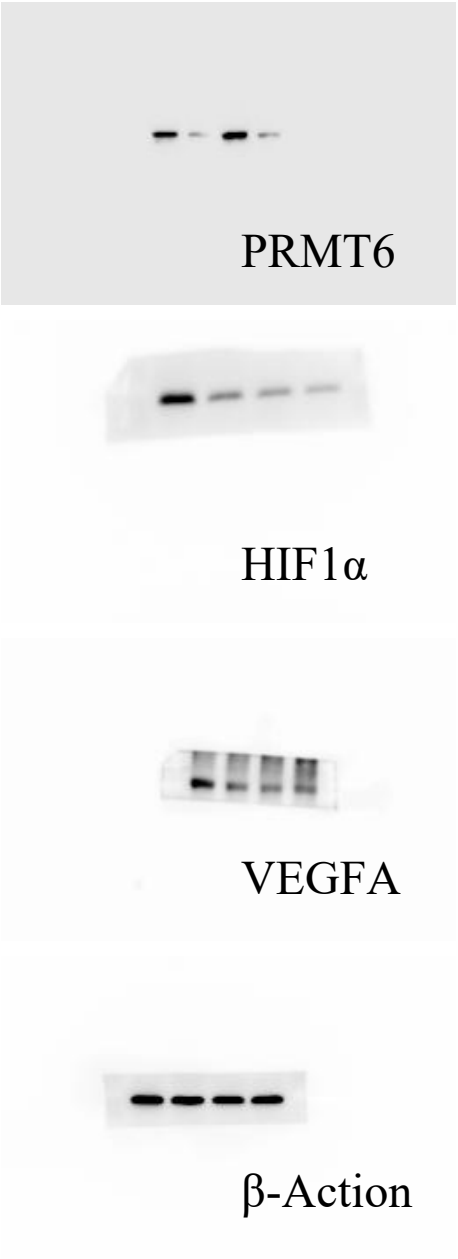

Figure 8G

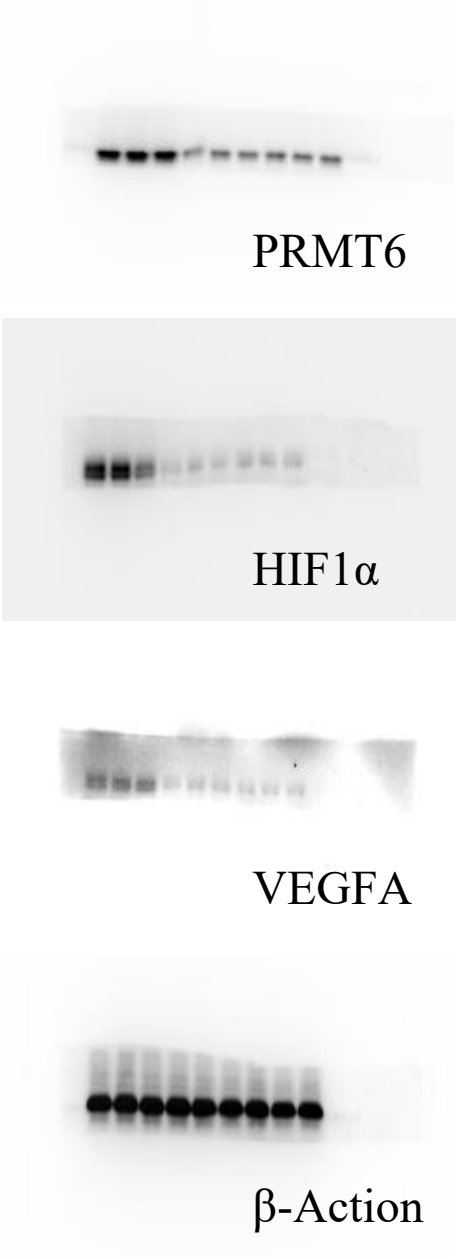

Supplement: Supplementary file 1 [file 41420_2026_3256_MOESM6_ESM.pdf]
