# Supplementary material for: PRMT6 acts as a pro-angiogenic factor in colorectal cancer
Source: Cell Death Discov. 2026 Jul 11;12:402. doi: 10.1038/s41420-026-03256-y (PMC13639044; doi:10.1038/s41420-026-03256-y)
Supplement: Supplementary file 2 [file 41420_2026_3256_MOESM1_ESM.docx]

**Supplementary figure legend**

**Supplementary figure legend 1**. **PRMT6 might function as a pro-angiogenesis factor in colorectal cancer**. A. Analysis of the statistical relationship between PRMT6 and VEGFA expression levels across different cancer types. B, C. Comparative statistical analysis of PRMT6 expression in colorectal cancer and corresponding normal colorectal tissues using data from the TCGA database. D. Statistical comparison of PRMT6 expression levels in colorectal cancer versus adjacent normal tissues based on the GSE24550 dataset; n = 13 (Normal), 77 (Tumor). E. Statistical evaluation of PRMT6 expression in cancer tissues compared to adjacent normal tissues using GEPIA database. F. ROC curve analysis to assess the efficacy of PRMT6 as a potential diagnostic marker for identifying colorectal tumors. Student's t-test was utilized for the statistical evaluation.

**Supplementary figure legend 2**. **Cancer patients with higher PRMT6 expression had shorter overall survival time**. Kaplan-Meier survival analysis for cancer patients grouped by PRMT6.

**Supplementary figure legend 3**. **PRMT6 positively regulated HIF1 signaling**. A. GSEA analysis was performed on various GEO gene sets. B. Western blotting was conducted to evaluate the expression of the targeted protein in shnc and shPRMT6 cancer cells under normoxic conditions. C. Western blotting was conducted to evaluate the expression of the targeted protein in shnc and shPRMT6 cancer cells under hypoxia conditions. D. Western blots were used to determine the protein expression in cancer cells that ectopically express either PRMT6 WT or the PRMT6 KLA mutant. E. Western blot analysis assessed the protein expression in cancer cells treated with either DMSO or PRMT6-IN-3. F, G. RT-PCR assays were conducted to measure the mRNA levels of HIF1α in different pre-treated cancer cell lines; n = 3. H. Representative images of immunohistochemistry (IHC) results are presented; scale bar = 50 µm. Statistical analysis was performed using Student's t-test or one-way ANOVA analysis. All western blot experiments were repeated three times.

**Supplementary figure 4**. **PRMT6-mediated HIF1α methylation blocked the interaction of HIF1α and TAX1BP1**. A. GENEMANIA database was used to assess the possibility of the interaction between PRMT6 and HIF1α. B. Western blotting and immunoblotting were utilized to evaluate the ADMA levels of HIF1α in cancer cells treated with either DMSO or PRMT6 inhibitors. C. Western blotting and immunoblotting were conducted to determine the ADMA levels of HIF1α in cancer cells subjected to hypoxia treatment compared to those not treated with hypoxia. D. Western blotting was conducted to determine the PRMT6 levels in cancer cells subjected to hypoxia treatment compared to those not treated with hypoxia. E. Knocking out HIF1α in LoVo and HCT116 using crisp-cas9 system. Western blots to confirm the successful construction. F. Constructing LoVo and HCT116 stably expressing HIF1α WT or HIF1α R463K. Western blots to confirm the successful construction. G. Using the CRISPR-Cas9 system, TAX1BP1 was successfully knocked out in LoVo cells, and subsequent Western blotting was performed to confirm the construction. H. Western blot and immunoblot analyses were conducted to investigate the interaction between HIF1α and TAX1BP1 in cancer cells that were transfected with either Vector, PRMT6 WT, or PRMT6 KLA mutant. All Western blot experiments were carried out in triplicate.
